# Mechanism of Ferroptosis and Its Role in Disease Development

**DOI:** 10.7150/ijbs.102859

**Published:** 2025-08-22

**Authors:** Zhangruijian Ye, Bin Xie, Yongguang Tao, Desheng Xiao

**Affiliations:** 1 Department of Pathology, School of Basic Medical Science, Xiangya School of Medicine, Central South University, Changsha, Hunan, China.; 2 Department of Pathology, Xiangya Hospital, Central South University, Changsha, Hunan, China.

**Keywords:** ferroptosis, cancer, lipid peroxidation, antioxidant system

## Abstract

Ferroptosis is a type of cell death that differs from general forms of cell death such as apoptosis. Iron accumulation and lipid peroxidation are distinct biochemical features of ferroptosis. Accumulation of iron ions in ferrodead cells can increase the probability of the Fenton reaction and produce more reactive oxygen species (ROS). Further, iron ions are cofactors for some intracellular oxidases. ROS, which are normally produced in the mitochondria, attack the phospholipid bilayer of the cell and produce lipid peroxides that ultimately kill the cell. Since the discovery of ferroptosis, its mechanism and relationship with diseases, such as nervous system diseases, tumors, and sepsis has been studied. Controlling disease development by regulating ferroptosis has thus become a popular topic of current research. This article summarizes the recent research progress on the mechanism of ferroptosis and its relationship with diseases. Overall, this study can provide a valuable reference for future ferroptosis studies.

## Introduction

**Figure 1:**
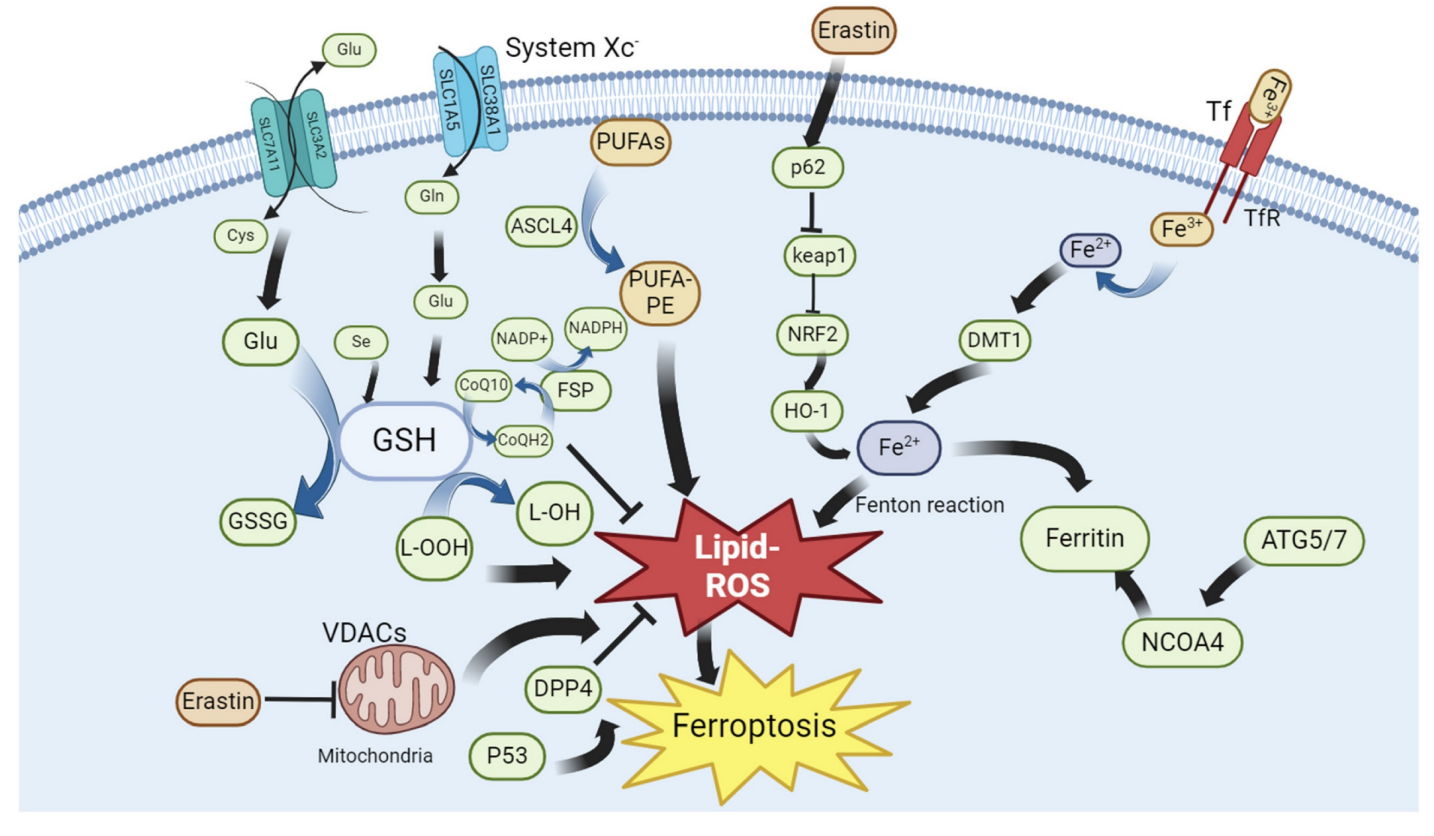
** Partial metabolic and cellular signaling pathways in ferroptosis:** Reactive oxygen species (ROS) production and lipid peroxidation are important biochemical features of ferroptosis. It is characterized by the accumulation of intracellular iron ions, which complement each other. Further, cells have various antioxidant systems, which together with lipid peroxidation constitute a dynamic balance and control the occurrence of ferroptosis.

Traditional cell death processes are well known, and new cell death processes have been discovered in recent years. These include ferroptosis, a type of cell death that is closely related to the metabolism of iron ions in the human body^1^. In 2012, ferroptosis was formally proposed to describe cell death different from that induced by RSL3^2^. Unlike general types of cell death, ferroptosis is a new type of programmed cell death, characterized by intracellular iron accumulation and uncontrolled intracellular lipid peroxidation. Because ferroptosis involves peroxide production, mitochondria are the organelles with the most obvious morphological (**Figure 1**) changes in ferroptosis. Loss of mitochondrial cristae, an increase in membrane density, and a decrease in mitochondrial membrane volume in ferroptotic cells have been observed by electron microscopy. Ferroptosis involves complex physiological and biochemical changes in lipid metabolism, iron homeostasis, and REDOX regulation. Ferroptosis occurrence and development are regulated by hundreds of enzymes. The ferroptosis pathway may differ in different organelles owing to the different oxidoreductases^3^. Recently, several studies have shown that ferroptosis is involved in the occurrence and development of several common clinical diseases, including liver cancer^4^, nervous system disease^5^ and ischemia/reperfusion injury^6^. Ferroptosis often plays key roles in the development and progression of these diseases. Inhibiting or promoting ferroptosis in these diseases may result in unexpected therapeutic effects. Therefore, this review summarizes the mechanisms and regulatory factors of ferroptosis and its relationship with various diseases. It aims to review previous research results and provide new directions for future research.

## Overview of Ferroptosis

Research on ferroptosis began with the discovery of the small-molecule erastin. The compound was first discovered in 2003. Cell death caused by this compound did not have the typical characteristics of apoptosis but belonged to a new form of cell death; erastin could selectively induce the death of cancer cells with high RAS expression through this pathway^7^. Erastin-induced cell death was non-apoptotic The caspase inhibitor BOCD-fmk failed to block erastin-induced cell death^8^. This new type of cell death was characterized by reactive oxygen species (ROS) accumulation, and was reversed by the iron chelator deferoxamine (DFO). Furthermore, erastin-induced ferroptosis was found to involve inhibition of the Xc system. The Xc system contains SLC7A11 and SLC3A2; SLC7A11 is primarily responsible for regulating Xc system activity 9. Subsequently, further revealed that erastin could function by acting on voltage-dependent anion channels (VDAC). VDAC is an ion channel located on the outer mitochondrial membrane that regulates the balance of ion entry and exit for the mitochondria^10^. Erastin reverses the inhibition of VDAC by tubulin, enhances mitochondrial metabolic activity, increases the ATP/ADP ratio, and increases ROS production^11^.

Erastin indirectly inhibits GPX4 activity and blocks GSH production, thus disturbing the balance between oxidative and antioxidant systems 12. Moreover, erastin blocks mitochondrial membrane permeability, thereby promoting cytochrome c release. Cytochrome c specifically promotes the lipid peroxidation of cardiolipin^13^. RSL3 is a well-known inducer of ferroptosis. RSL3-induced ferroptosis is characterized by the inhibition of the GPX4 system in cells, whereas the overexpression of GPX4 can rescue RSL3-induced ferroptosis. The diversity of ferroptosis inducers indicates the complexity of ferroptosis regulation and the existence of multiple ferroptotic pathways in cells. Therefore, the occurrence and development of ferroptosis can be controlled through many aspects. In addition to erastin, many other drugs are capable of inducing ferroptosis in cells, including iron-based nanodrugs (NDs). In clear cell renal cell carcinoma (ccRCC), iron-based metal-organic framework nanoparticles (MIL-101(Fe) NPs) can deliver RSL3 into cancer cells. Subsequently, RSL3 and iron ions are released in an acidic environment, which promotes ferroptosis of cancer cells 14. A drug named GSK-J4 was identified through the integrated screening of small-molecule inhibitor libraries and pharmaceutical-targeting CRISPR libraries. Donafenib, in combination with GSK-J4, has been shown to be useful in cancer treatment. The two drugs synergistically promote HMOX1 expression and subsequently increase the intracellular free iron ion levels, leading to ferroptosis in hepatocellular carcinoma (HCC) cells^15^. Further, tagitinin C, isolated from mangosteen, inhibits the growth of colorectal cancer cells and promotes ferroptosis in HCT116 cells. Specifically, tagitinin C increases intracellular iron levels by upregulating heme oxygenase-1 (OH1); notably, tagitinin C and erastin exhibit a synergistic effect on cancer cell ferroptosis^16^. Sorafenib induces ferroptosis, and its effect on ferroptosis in gastric cancer cells strongly correlates with ATF2 expression. When sorafenib induces ferroptosis, ATF2 is activated and acts on HSPH1. The latter increases the stability of SLC7A11. HSPH1 knockout promotes sorafenib-induced ferroptosis in gastric cancer cells^17^. Quercetin, a ketone compound, promotes ferroptosis in gastric cancer cells by increasing intracellular ROS and iron ion concentrations and decreasing antioxidant protein levels via SCL1A5 inhibition. Quercetin can activate p-Camk2, lead p-DRP1 upregulation, inhibit the Nrf2/xCT system, and eventually induce ferroptosis in gastric cancer cells^18^. Cannabidiol exhibits significant cytotoxicity against glioma cells. It induces ERK activation, increases intracellular ROS levels, and promotes lipid peroxidation in cancer cells. It promotes the expression of autophagy-related genes such as LC3II and Atg7 and reduces GSH levels. Eventually, autophagy or ferroptosis occurs in cancer cells. Cannabidiol-induced autophagy and ferroptosis can be partially reversed by an ERK inhibitor, indicating that cannabidiol induces ferroptosis in cancer cells via the ERK pathway^19^.

## Biochemical Characteristics of Ferroptosis

### Iron ion accumulation

**Figure 2:**
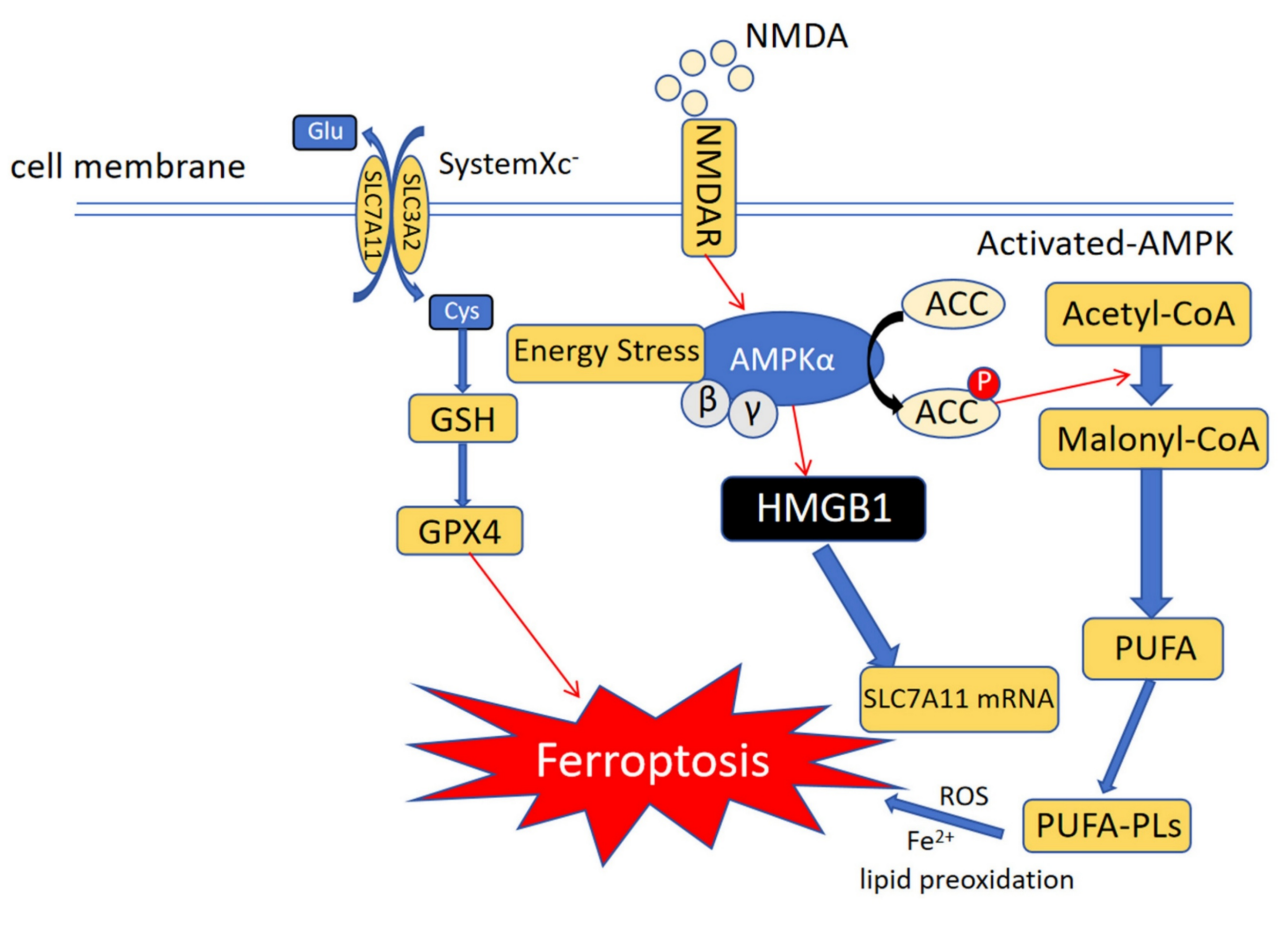
** Major pathways of iron metabolism in cells:** Extracellular Fe^3+^ is recognized by Tf and TfR1 and transported into the cell, where it is converted from STEAP3 to Fe^2+^ within the endosome and transported by DMT1 to the cytoplasm. Extracellular Fe^3+^ can also be converted from Cyt b to Fe^2+^ and transported into the cell by DMT1 or ZT. Intracellular Fe^2+^ and Fe^3+^ enter the labile iron pool (LIP), and these iron ions undergo the Fenton reaction to promote ferroptosis or combine with ferritin. Finally, iron ions are expelled from the cell via FPN1.

The chemical properties of iron ions explain why iron is required for ferroptosis (**Figure 2**). Iron ions exhibit unique REDOX properties (switching between Fe^2+^ and Fe^3+^), allowing them to play a special role in various biochemical reactions^20^. For example, iron ions can be used as coenzymes or key components of cytochromes, hemoglobin, and myoglobin^21^. Simultaneously, Fe ions catalyze free radical production from peroxides. If not reduced in time, these free radicals attack cell membranes, organelles, DNA, and other cellular contents, resulting in oxidative damage^22^. Iron ions play a central role in ferroptosis as part of various oxidoreductases and the Fenton reaction with hydrogen peroxide^23^. In the human body, iron ions cause major pathological damage, mainly as non-transferrin-bound iron (NTBI). In addition to NTBI, the unstable iron pool (TIP) can generate ROS to attack cells^24^. Intracellular ferritin can be used as a defense mechanism against iron ion-induced cellular damage. Specifically, ferritin functions as a ferroxidase, converts Fe^2+^ to Fe^3+^, stores iron ions, and sequesters iron ions from the cytosolic environment. Ferritin also plays an important role in cell survival. Ferritin H knockout is lethal in mice^25^. Ferritin plays an important role in preventing cardiomyopathy occurrence and development. A lack of ferritin leads to an increase in the intracellular free iron concentration and ROS accumulation, making cardiomyocytes more susceptible to ferroptosis^26^. The iron response mediated by transferrin receptor (TFR) plays a key role in doxorubicin (DOX)-induced apoptosis^27^. The use of nanomaterials to target ferroptosis is popular in the treatment of various diseases. Different nanomaterials can be designed to meet the requirements for treating various diseases. Moreover, nanomaterials can distinguish cancer cells from normal tissues by recognizing their hypermetabolic characteristics or preventing ferroptosis in normal cells by activating antioxidant pathways. In recent years, a new nanomaterial, F-bio-HJ, has been developed to address the high wound infection rates and difficult self-healing in patients with diabetes. This material can protect normal cells by targeting bacteria to induce ferroptosis. In addition, Fe_2_O_3_, a common type of iron oxide, is used as a free iron donor. The ability of F-bio-HJ to protect wounds was found to be significantly reduced in the presence of the iron chelator EDTA. Therefore, one of the antibacterial mechanisms of F-bio-HJ is the production of Fe^3+^ in acidic environments, leading to excessive lipid peroxidation in cells and irreversible death. As a component of the antioxidant system, GPX4 expression continued to decrease and the free iron level continued to increase after F-bio-HJ treatment, thus rapidly inducing ferroptosis. Normal cells can resist F-bio-HJ-induced ferroptosis because of their normal antioxidant systems. Moreover, F-bio-HJ can rapidly deplete extracellular glucose, which induces energy stress in normal cells and activates the AMPK pathway, an important pathway for resisting ferroptosis. The protective effect of F-bio-HJ on normal cells is mainly mediated through the AMPK pathway. In summary, F-bio-HJ is an ideal new anti-infection nanomaterial that promotes tissue repair via ferroptosis^28^. Overall, Fe ions play a complex and important role in ferroptosis.

### Lipid peroxidation

Lipids are the components and regulatory sites of various organelles in cells, as well as targets of various reactive substances, such as reactive oxygen species (ROS) and reactive nitrogen species (RNS). Lipid peroxidation is a process in which active substances act on various lipids in cells 29. Lipid peroxidation is associated with various types of cell death. Ferroptosis is closely related to lipid peroxidation and is characterized by increased iron-induced lipid peroxidation^30^. The Fenton reaction is an important process in the production of active substances in cells. Two important reactive radicals involved in lipid peroxidation are hydroxyl radical (OH·) and hydroxyl peroxide radical (OOH·). Under the catalysis of peroxidases (ALOXs), reactive substances interact with polyunsaturated fatty acids (PUFAs) on the membrane of organelles to produce reactive lipids (RLS), such as malondialdehyde (MDA), which in turn activate ferroptosis^31^. Adenosine 5'-monophosphate (AMP)-activated protein kinase (AMPK) is a central regulator of the body that responds to stress. AMPK is activated in response to energy stress. It inhibits ferroptosis by inhibiting the synthesis of PUFAs and other fatty acids and by activating Nrf2^32^, ^33^. Further, monounsaturated fatty acids (MUFAs) inhibit caspase-dependent fatty acid lipotoxicity. MUFAs are competitive inhibitors of PUFAs synthesis. This may explain why MUFAs protect against atherosclerotic heart diseases. Exogenous MUFAs inhibit the apoptotic lipotoxicity caused by the accumulation of saturated fatty acids (SFAs). Therefore, MUFAs are regulators of apoptosis and ferroptosis. In conclusion, a balance between PUFAs and MUFAs is necessary for the occurrence and development of normal cell ferroptosis^34^. Further, lipid peroxidation is dependent on peroxisomes (LOXs). Recently, ALOX15, a member of the LOX family, was found to promote ferroptosis in HT1080 cells by generating peroxides. A specific inhibitor of ALOX15 significantly reduced ferroptosis induced by erastin and RSL3. Through CRISPR/Cas9 screening, cytochrome P450 oxidoreductase (POR) was also found to be a key mediator of ferroptosis. Moreover, its ferroptosis-promoting effects are observed in many cancer types. Studies have shown that POR induces cell membranes composed of polyunsaturated phospholipids, eventually leading to ferroptosis 35. Verteporfin (VP), a photosensitizer, can significantly increase ROS and lipid peroxide levels in pancreatic ductal cancer (PDAC) cells, independent of the Hippo-YAP pathway. Notably, this effect is reversed by ferrostatin-1 or liproxstatin-1. These results suggest that VP may be a novel therapeutic option for treating PDAC^36^. In recent years, the content of diacyl-containing ^PUFAs^ (PC-PUFA2s) has been found to increase significantly *in vivo* after fatty acid or phospholipid treatment. Compared with PCS containing a single PUFA tail (PC-PUFA1s), PC-PUFA2s are more potent at inducing ferroptosis. This change is associated with an increased sensitivity of cancer cells to ferroptosis. Supporting the conclusion that PC-PUFA2s are strongly associated with ferroptosis, the abundance of PC-PUFA2s in ferroptosis-sensitive cells was significantly higher than in ferroptosis-insensitive cells before treatment with ferroptosis-inducing agents. Further, PC-PUFA2S-treated cells showed a significant increase in the free PUFA content, and PC-PUFA1s co-treatment in Calu-1 cells showed a high synergistic effect. These results suggest that PUFA and PC-PUFA1s, as precursors of PC-PUFA2s, are likely involved in the development of ferroptosis^37^. SC5D overexpression and DHCR7 knockout reduced the sensitivity of cells to ferroptosis during cholesterol synthesis. The intermediate, 7-DHC, was shown to protect cells from ferroptosis. This mechanism involves the conjugated double bond of the B-ring of 7-DHC. Ergosterol, a structurally similar D-sterol, also exhibits antiferroptotic activity. This structure may prevent ferroptosis by diverting the damage caused by oxygen radicals from the phospholipid to its internal core. This study also confirmed that the production of phospholipid peroxides is an inevitable step in ferroptosis^38^. In summary, lipid peroxidation plays an irreplaceable role in ferroptosis. Lipid peroxides can attack various biological membranes inside cells, such as the lysosomal membrane, causing further damage to cells undergoing ferroptosis. Notably, most existing antiferroptosis drugs can regulate lipid peroxidation.

### Relationship between iron accumulation and lipid peroxidation

Ferroptosis is typically characterized by iron accumulation and lipid peroxidation. Iron ions are the basic cofactors for REDOX reactions in cells. If intracellular iron is not properly regulated, the balance of intracellular REDOX reactions is destroyed; therefore, the level of iron in the cells must be strictly controlled^39^. Although Fe^3+^ is highly oxidative, it is also a coenzyme for many cellular oxidases. Therefore, when accumulation of iron ions in the labile iron pool of the cell increases, the intracellular REDOX balance is disrupted, as non-transferrin-binding proteins are bound to iron production. A large amount of free iron ions is released under the action of nuclear receptor activator 4 (NOCA4), which initiates the Fenton reaction^40^. Consequently, several peroxidases (ALOXs) are generated. These oxidative free radicals attack intracellular PUFAs to produce RLS and induce ferroptosis. Lipofuscin is a specialized cellular structure found in cardiomyocytes. In H9c2 cardiomyocytes, lipofuscin induces ROS generation to accelerate cardiomyocyte aging. The addition of exogenous iron ions to cardiomyocytes induces lipid peroxidation and ferroptosis 41. Transmission electron microscopy (TEM) results indicate that mitochondrial vacuolization is closely related to lipofuscin accumulation in brain cells. Antioxidants such as pantothenic acid and vitamin E prevent lipid peroxidation and iron accumulation in cells^42^. Lipid reactive oxygen species (ROS) accumulation and ferritin deposition are increased in mice fed a methionine/choline-deficient diet (MCD). The ferritin deposition inhibitors Fer-1, Lip-1, and enoyl-CoA hydratase 1 (ECH1) significantly reduce the severity and tissue damage of NASH in mice^43^. These findings suggest a significant relationship between ferroptosis and lipid peroxidation.

## Other Components

### The relationship between ferroptosis and mitochondria

Ferroptosis is closely related to the mitochondria. Mitochondrial dysfunction can lead to increased intracellular ROS production, which is an important cause of endothelial cell dysfunction^44^. Therefore, drugs that maintain mitochondrial homeostasis can inhibit ferroptosis in normal cells (such as cardiomyocytes and vascular endothelial cells), mitochondrial dysfunction is an important indicator of cardiovascular disease^45^. Therefore, there is an urgent need to identify drugs that inhibit ferroptosis in normal cells by inhibiting mitochondrial dysfunction. Studies have shown that quercetin can effectively protect cardiomyocytes. Mechanistic studies have shown that quercetin can reduce the myocardial cell damage caused by mitochondrial dysfunction through DNA-PKcs and regulate mitophagy^46^. Mitochondrial fission factor (Mff) mediates mitochondrial fission in cardiac ischemia-reperfusion injury. NR4A1, a molecule that regulates apoptosis under stress conditions, is activated under ischemia-reperfusion. NR4A1 knockout mice show enhanced resistance to ischemia-reperfusion injury, suggesting that the Nr4a1-Mff axis is a potential target for resistance to ischemia-reperfusion injury and ferroptosis in cardiomyocytes^47^. Recently, several studies have shown that mitochondrial dysfunction is closely associated with disease occurrence and development. For example, Tongyang Huoxue decoction (TYHX) protects sinoatrial node cells (SNC) by stabilizing the stability of β-tubulin and sirt1 through VDAC1 to inhibit mitochondrial stress and excessive SNC division. Similarly, TYHX can activate SIRT1 to protect mitochondrial function. Thus, TYHX can effectively alleviate oxidative damage in sinoatrial node cells under hypoxic stress, and its underlying mechanism may be closely related to the protection of mitochondrial function and regulation of mitochondrial quality surveillance (MQS), thereby indirectly inhibiting ferroptosis in sinoatrial node cells^48^, ^49^. Similar to TYHX, Zishenhuoxue decoction (ZSHX) also exerts protective effects on cardiovascular endothelial cells by increasing TMBIM6 expression and regulating mitochondrial calcium homeostasis and MQS abnormalities through the VDAC1-TMBIM6 axis^50^. Hematoxylin A (SA) regulates ferroptosis in non-small cell lung cancer (NSCLC) cells through the Nrf 2/GPX 4/xCT axis and regulates mitochondrial function. Nrf2 knockdown affects the therapeutic effects of SA. SA also affects mitochondrial function in tumor cells by inhibiting FUNDC1 phosphorylation and downregulating TOM20 expression. Therefore, SA is a potential drug for treating NSCLC^51^. Mitochondria act as stress sensors in cells. Under some pathological conditions, mitochondrial function is prone to disturbances, leading to ROS production, which mediates ferroptosis. Mitochondria also affect the stability, structure, and function of vascular endothelial cells. Impaired mitophagy is associated with endothelial cell dysfunction^52^. Opening of the mitochondrial permeability transition pore and calcium overload are also closely associated with the occurrence and development of ischemic cardiomyopathy^53^. Ginsenoside Rb1 targets dual-specific phosphatase 1 (DUSP 1), which regulates mitochondrial activity. Under stress conditions, ginsenoside Rb1 regulates the DUSP 1, TMBIM 6 and VDAC1 axis to regulate the mitochondrial function of cardiomyocytes, inhibit the inflammation development, and indirectly inhibit ferroptosis in cardiomyocytes^54^. During ferroptosis, the mitochondria shrink with an increase in membrane density, leading to dramatic changes in mitochondrial viscosity. A newly developed mitochondria-targeting probe, TJ-FRP, used for fluorescence imaging to measure cell viscosity, can be used to detect changes in the mitochondria during ferroptosis^55^. In recent years, mitochondria-related genes (such as FBXO7, PGS1, LYRM7, and BCS1L) have been found to be closely related to immune cell infiltration in septic cardiomyopathy, and these genes are also closely related to mitochondrial dysfunction. Ferroptosis occurrence and development in cardiomyocytes is a result of mitochondrial dysfunction^56^. Mitochondria are widely recognized as the major sites of biological metabolism, oxidative phosphorylation, and ATP production^57^, and are also prone to ROS production. Timely removal of ROS produced by mitochondria helps delay the progression of cellular senescence^58^. Studies have shown that almost all regulated cell deaths (RCDs) involve the mitochondria. Mitochondria are also involved in the development and regulation of ferroptosis^59^. Induction of ferroptosis by the classical ferroptosis activator erastin or cysteine starvation was found to be attenuated in CCCP-treated cells. This suggests that mitochondria may contribute to cysteine deprivation-induced ferroptosis^60^. Characteristic changes in mitochondria, such as increased inner membrane density, loss of mitochondrial cristae, and mitochondrial atrophy have been observed in almost all ferroptotic cells^61^. The classical ferroptosis activator erastin induces ferroptosis by targeting the mitochondrial voltage-dependent anion channel (VDAC). VDAC acts as a protein channel that facilitates ion transport across the membrane. There are two isoforms, VDAC2 and VDAC3, located above the mitochondria. Erastin inhibits VDAC function, leading to ROS accumulation in mitochondria^62^. Simultaneously, VDAC1 binding protein V-Set and transmembrane domain containing 2-like protein (VSTM2L) are positively correlated with prostate cancer (PCa) progression and are key regulators of ferroptosis^63^. Moreover, ROS can accumulate in cells lacking mitochondrial function. Mitoquinone (MitoQ), a mitochondria-targeted ROS scavenger, inhibits ferroptosis by scavenging ROS from mitochondria without damaging the mitochondrial membrane^64^. FUN14 domain-containing 2 (FUNDC2) is a mitochondrial membrane protein that interacts with SLC25A11 to regulate mitochondrial GSH (mitoGSH) and ferroptosis. This regulatory system is referred to as the FUNDC2-SLC25A11 axis, which plays a protective role in myocardial injury induced by Adriamycin (DOX)^65^. Ceramide kinase (CERK) is an enzyme present in the mitochondria and plasma membrane of mammalian cells. CERK functions in the survival maintenance of KRAS-mutant NSCLC CERK regulates mitochondrial membrane potential (MMP) and reduces ROS levels in cancer cells. Interestingly, pretreatment of A549 cells with antioxidants reverses the effects of CERK inhibition on tumor cells and the expression of the ferroptosis marker TfR1^66^. In doxorubicin-induced cardiomyopathy (DIC), GPX4 expression is significantly decreased in diseased cardiomyocytes. The MDA and mitochondrial LPs increase in a DOX concentration-dependent manner. Thus, mitochondria-dependent ferroptosis plays a key role in DIC^67^. Another mitochondrial inner membrane enzyme, GPD2, is resistant to RSL3-induced ferroptosis. Notably, Gpd2-depleted cells are not sensitive to Fin56-induced ferroptosis, and FIN56 is CoQ-dependent. The mechanism by which GPD2 protects against ferroptosis involves oxidation of G3P to DAP and reduction of CoQ to CoQH2 at the inner mitochondrial membrane^68^. Cyclic AMP-GMP synthase (cGAS) was recently found to possess two mitochondrial binding sites. Further studies have shown that cGAS in the mitochondria of tumor cells, such as Hep3B and PLC cells, could resist ferroptosis in tumor cells. Overexpression of MTS-cGAS promoted tumor cell growth, and treatment of cells with a DRP1 inhibitor abolished this tumor growth-promoting effect^69^. The small molecule CGI1746 was identified by screening a kinase inhibitor library. It is a potent ferroptosis inhibitor. The mechanism by which CGI1746 inhibited ferroptosis was found to be its chaperone, sigma-1R (σ1R), a receptor located on the mitochondrial membrane. Inhibition of σ1R protects mice against ferroptosis-induced renal injury in acute kidney injury (AKI)^70^. Mitochondria are the center of metabolism for various substances in the cell, making it easy to produce reactive oxygen species and attack lipids to cause lipid peroxidation. In contrast, many antioxidant enzymes are present in the mitochondrial membrane. Recent studies have shown that Icariin (ICA) induces ferroptosis in colorectal cancer cells by inducing mitochondrial dysfunction. Simultaneously, ICA can cooperate with anti-PD-1 to promote CD8^+^ T cells to secrete IFN-γ and promote anti-tumor effects^71^. In summary, mitochondrial dysfunction leads to disruption of the intracellular REDOX balance, leading to a large amount of intracellular ROS and promotion of cell ferroptosis. Meanwhile, pathological states such as the disappearance of mitochondrial cristae can be observed in cells undergoing ferroptosis; therefore, ferroptosis is closely related to mitochondrial dysfunction. In future, more drugs targeting the mitochondria to regulate ferroptosis are expected to be discovered.

### Iron metabolism in ferroptosis

Iron is an essential element in the human body. There are two forms of iron in the body, Fe^2+^ and Fe^3+^. Most iron in the body is stored as hemoglobin. The iron transporter on the cell membrane is a divalent metal transporter (DMT1) that is finely regulated by the iron requirements of the body. DMT1 expression is elevated in iron-deficient animals^72^. DMT1 plays a key role in temozolomide-induced ferroptosis in glioblastoma. DMT1 knockdown by siRNA inhibits the temozolomide-induced decrease in cell viability^73^. Transferrin (TF) is an important iron-binding protein mainly synthesized in the human liver^74^. In ferroptosis-induced liver fibrosis, iron produced by the decomposition of free heme is the main cause of hepatocyte death. SLC39A14 can transfer free iron from the extracellular to intracellular compartment in the liver cells of TF-lko mice. These results suggest that SLC39A14 is a potential therapeutic target for ferroptosis-induced liver fibrosis^75^. The non-TF-bound iron (NTBI) content increases when TF-bound iron exceeds the upper limit. In general, NTBI is considered a major cause of excess iron-related diseases. Lactotransferrin (LTF) is secreted by several organisms. It binds iron ions and has antibacterial, anti-inflammatory, and anti-cancer effects^76^. In neuronal post-stroke hyperglycemic mice, LTF expression in inflammation-infiltrating neutrophils is decreased in streptozotocin (STZ)-treated mice. STZ-treated mice are more susceptible to ferroptosis of neurons and a decline in neurological function^77^. The oxidoreductase system in the body reduces Fe^3+^ to Fe^2+^ through the reducing enzyme, cytochrome b reductase 1 (CYBRD1). It also mediates the regulation of iron ions *in vivo*^78^. Generation of ferrous ions (Fe^2+^) induces the production of intracellular reactive oxygen species (ROS) and DNA damage. CDGSH iron-sulfur domain 2 (CISD2) plays an important role in regulating intracellular iron levels. It is also involved in oxidative stress^79^. Previous studies have shown that CISD21 knockdown aggravates erastin-induced ferroptosis. Specifically, CSID2-mediated ferroptosis is associated with autophagy-dependent Nrf2 inhibition. Decreased p62 phosphorylation results in decreased p62 binding to Keap and increased Nrf2 degradation in CSID2-KO cells^80^. Ferritin heavy chain 1 (FTH1) plays an important role in the homeostatic regulation of iron storage during ferroptosis^81^. FTH1 may also be involved in ferritin autophagy. Ferritin autophagy produces additional substrates for ferroptosis^82^. CoQ, a product of the mevalonate pathway, which is responsible for the synthesis of cholesterol and other important biomolecules in the body, can act as an electron carrier in mitochondrial REDOX reactions. CoQH2, the reduced form of CoQ, is a key antioxidant *in vivo*^83^. MicroRNA-612 (miR-612) regulates ferroptosis in hepatocellular carcinoma cells through the hydroxyacyl-CoA dehydrogenase alpha subunit (HADHA). Further, knockdown of miR-612 or overexpression of HADHA in Huh7 cells significantly increases the expression levels of MVA pathway-related proteins. This further supports the role of MVA-related pathways in ferroptosis^84^. Intracellular ROS levels are significantly increased during baicalin-induced ferroptosis. Intracellular ROS level in FTH1 transfected cells was significantly higher than that in untransfected cells. These results suggest that FTH1 plays a key role in baicalin-induced ferroptosis^85^. Nuclear receptor coactivator 4 (NCOA4) recognizes and binds to FTH1 in cells, inducing lysosomal hydrolysis and free iron release. NCOA4 knockdown significantly reduces the amount of intracellular free iron. Compound 9a inhibits the interaction between NCOA4 and FTH1 by binding to NCOA4383-522 and attenuates ferroptosis^86^. Further, mitochondrial ferritin (FTMT) is regulated by NCOA4. FTMT synergizes with FTH to protect macrophages from RSL3-induced ferroptosis. However, in HT1080 cells, NCOA4 is not sensitive to hypoxia; therefore, FTMT does not change significantly^87^. The efflux of excess iron from the cell is mediated by solute carrier family 40 member 1 (SLC40A1), also known as FPN1. To date, SLC40A1 is the only iron efflux protein identified. Reduced SLC401 activity can lead to intracellular iron accumulation and eventually ferroptosis^88^. Supplementation with mesenchymal stem cells (MSCs) in a mouse model of acute liver failure (ALF) can prevent ferroptosis in hepatocytes. Mechanistic studies have shown that MSCs can downregulate hepcidin and upregulate SLC40A1 levels, thereby reducing the possibility of ferroptosis by reducing iron ion deposition in hepatocytes^89^. In the hippocampus of diabetic rats, SLC40A1 expression in ferroptotic cells is significantly decreased, whereas the levels of 4-HNE and MDA are increased. This suggests that SLC40A1 is involved in the cognitive dysfunction of patients with diabetes^90^. In HK-2 cells treated with 2',7 '-dichlorofluorescein diacetate (DA), SLC40A1 ubiquitination was increased, SLC40A1 function was decreased, and iron efflux was decreased. However, it did not affect the entry of iron into the cell or the storage and utilization of iron within the cell. However, this effect was significantly reversed by treatment with dapagliflozin. This reduced the ubiquitination of SLC40A1^91^. Two binding sites of microRNA-147a were identified in the 3'-UTR of SLC40A1. Moreover, miR-147a mimics significantly reduced SLC40A1 expression in U87MG and A172 cells to induce ferroptosis in glioblastoma cells^92^. The concentration of iron ions in cells is directly related to ferroptosis. The intracellular iron concentration is controlled by pathways that mediate the entry of iron into the cell as well as the expulsion of iron from the cell; therefore, targeting these pathways to control ferroptosis in cancer is critical.

### Nuclear erythrocyte 2 related factor 2 (Nrf2) Pathway

Nrf2 is a major molecule that regulate REDOX status in the body^93^. It has been reported to affect almost all anti-ferroptosis pathways. These include, but are not limited to, nicotinamide adenine dinucleotide and GPX4 pathways. Nrf2 is sensitive to intracellular ROS and RNS concentrations. Once ROS and RNS exceed a specific threshold, Nrf2 induces ARE gene action, which rapidly controls excess ROS and RNS to safe levels. Therefore, Nrf2 is indispensable for maintaining REDOX stability in the body^94^. Nrf2 regulates cellular antioxidant functions. Nrf2 knockout (KO) mice are more susceptible to cisplatin-induced AKI Motherwort reverses cisplatin-induced liver and kidney damage, and this protective effect is exerted by activating Nrf2 and inducing antioxidative damage^95^. IKK/IκB/NF-κB activation is strongly blocked in erastin-exposed HT22 cells transfected with Nrf2siRNA. Compared with control APP/PS1 mice, forsythoside A treatment was found to significantly reduce the incidence of ferroptosis and neuroinflammation, and this effect was achieved through the Nrf2 pathway^96^. Further, Nrf2 indirectly controls ferroptosis by regulating iron metabolism. It regulates the content, destruction, and resynthesis of hemoglobin via iron chelatase (FECH). It is also indirectly involved in iron storage and transfer^97^. Nrf2 is also associated with GCH1. It can upregulate GCH1 activity and increase BH4 synthesis, which increases the ability of cells to resist oxidation. Saikosaponin A can protect against mastitis caused by *S. aureus* through activation of the SIRT1-Nrf2 pathway. However, this effect is reversed by a SIRT1 inhibitor^98^. Compared to WT cells, the mRNA level of vesicle-associated membrane protein 8 (VAMP8) was significantly decreased in Nrf2-knockdown cells. A positive correlation was observed between VAMP8 and Nrf2 expression levels. VAMP8 knockdown significantly increased cellular NCOA4 and FTH1 expression. Taken together, these studies revealed a novel Nrf2-mediated pathway for ferroptosis^99^. Piceatannol (PCT), a natural stilbene compound, can prevent myocardial ischemia-reperfusion injury owing to its significant antioxidant effects, and prevent myocardial cell damage caused by ferroptosis. Further studies have shown that PCT significantly upregulates the expression of Nrf2. After PCT treatment, the level of Fe^2+^ decreased, FPN1 expression increased, and TfR1 expression was inhibited. These results suggest that PCT most likely inhibits ferroptosis via the Nrf2 pathway. Further, some studies have found that a reduction in estrogen can alter the level of iron metabolism in the body, thereby inducing menopausal osteoporosis (PMOP). Lack of estrogen increases the possibility of ferroptosis in osteocytes. Nrf2 is a key regulator of ferroptosis in osteocytes. Nrf2 regulates the expression of nuclear factor κb ligand (RANKL) by regulating DNA methyltransferase 3a (Dnmt3a)-mediated DNA methylation levels at the RANKL promoter. This is an important ferroptosis mechanism in osteocytes. This suggests that osteocyte ferroptosis is involved in the development of PMOP^100^. As Nrf2 acts as a major antioxidant during ferroptosis, it may be clinically targeted to develop anti-cancer drugs. In conclusion, Nrf2 could attenuate ferroptosis incidence and enhance the antioxidant capacity, anti-ferroptosis, and anti-inflammatory effects in cells.

### AMP-activated protein kinase (AMPK) Pathway

**Figure 3:**
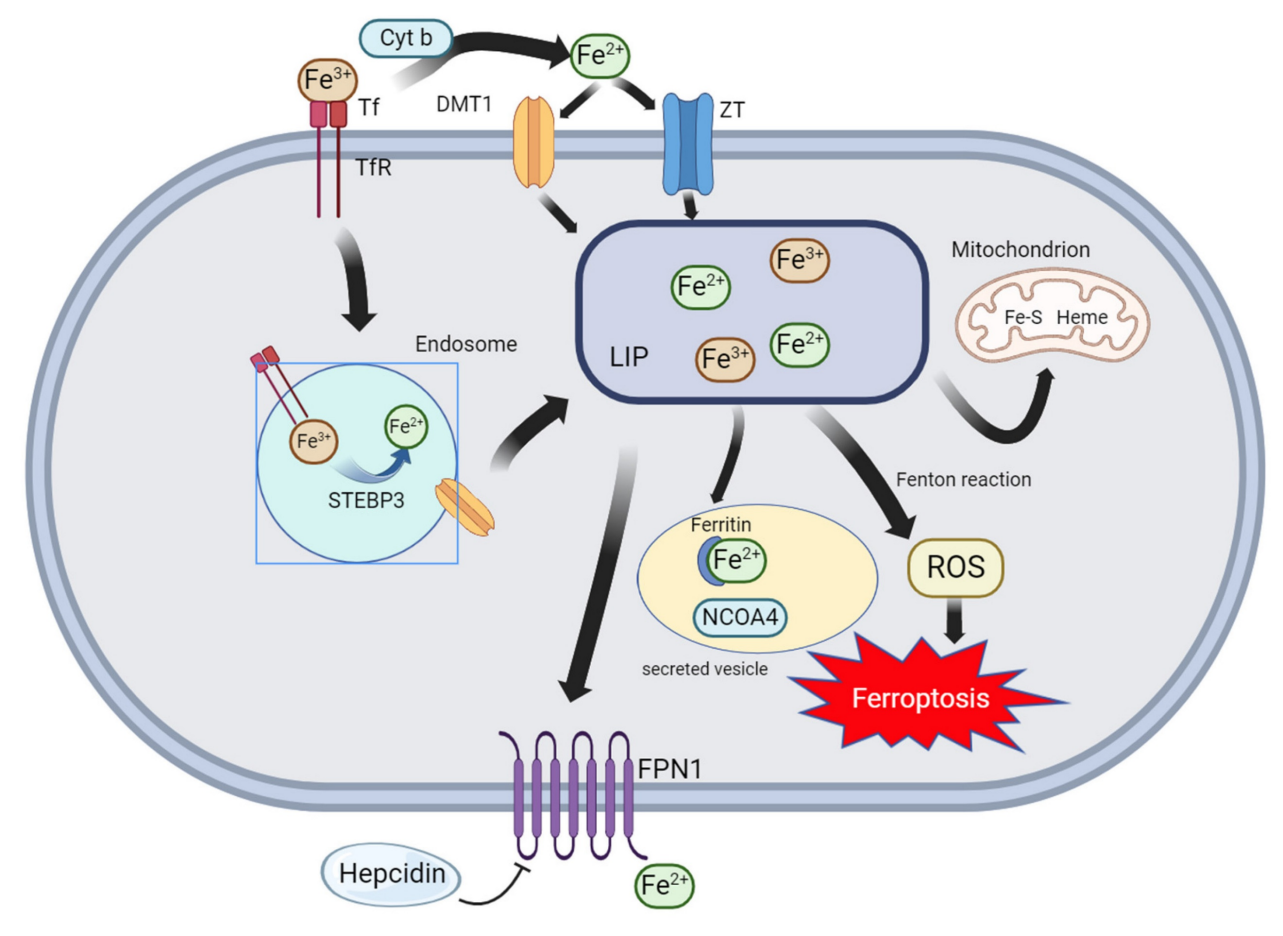
** Ferroptosis and immunity: Role of AMPK-mediated energy stress in ferroptosis:** During ferroptosis, energy stress or NMDAR activates the AMPK pathway and increases SLC7A11 mRNA transcription to resist ferroptosis. Similarly, AMPK can block PUFA synthesis and prevent ferroptosis from occurring via another route, such as epigenetic regulation.

The AMP-activated protein kinase (AMPK) pathway is responsible for energy balance regulation in eukaryotic cells and a target of many therapeutic drugs (**Figure 3**). The primary intracellular function of AMPK is to monitor ATP level changes and combine them with the phosphorylation of downstream substrates, leading to an increase in the rate of ATP production 101. AMP-activated protein kinase (AMPK) plays an important role in maintaining energy balance and regulating metabolic stress. Glucose-starved mice were found to be resistant to erastin-induced ferroptosis. This suggests that AMPK significantly reduces the possibility of energy stress-induced ferroptosis. Furthermore, AMPK expression negatively correlated with ferroptosis in SCL7A11-expressing cells^102^. The LKB1-AMPK axis inhibits ACC1, a key enzyme in fatty acid biosynthesis, under conditions of energy deficiency. Ferroptosis strongly induces ACC1 phosphorylation in wild-type mouse embryonic fibroblasts, but not in AMPK knockout cells^103^. The Forkhead box O3a (FoxO3a)-AMPK axis regulates activation of glucose starvation and inhibits erastin-induced ferroptosis. Phosphorylation of FoxO3a is upregulated during erastin-induced ferroptosis under energy stress. Interestingly, FoxO3a knockdown in MCF-7 cells restored sensitivity to erastin and increased intracellular ROS levels, but did not restore RSL3-induced ferroptosis sensitivity^104^. N-Methyl-d-aspartate receptors (NMDARs) are classical voltage-gated channels that activate ferroptosis in vascular endothelial cells (VECs) following NMAD or GLU treatment. This activation is regulated by the PP2A-AMPK-HMGB1 axis. Simultaneously, the intracellular levels of HMGB1 was significantly increased in VECs treated with NMAD or GLU. After HMGB1 knockdown, the cell death induced by these two compounds was significantly inhibited. Further, the NMDAR-selective inhibitor MK-801 significantly inhibited RSL3-or erastin-induced ferroptosis. These results suggest that NMDARs are involved in the ferroptosis of VECs induced by rsl3 or erastin via the PP2A-AMPK-HMGB1 pathway^105^. Quercetin (QCT) plays an important role in osteoarthritis occurrence and development, and can significantly improve the destruction and degradation of articular cartilage. QCT promotes the expression of p-AMPK, thereby inhibiting the ferroptosis of articular chondrocytes and playing a cytoprotective role^106^. Similarly, tetramethylpyrazine (TMP) can effectively inhibit the occurrence and aggravation of cerebral ischemia-reperfusion injury by reducing the levels of Fe^2+^, 4-hydroxynonenal, malondialdehyde, and acyl-CoA synthetase long-chain family member 4. TMP also significantly increases p-AMPK levels, thereby inhibiting cellular ferroptosis 107. In conclusion, AMPK is another key target in regulating ferroptosis.

## Antioxidant System

The cellular antioxidant system can control the various active substances produced in cells to a certain level^108^. Ferroptosis is closely related to the imbalance in the oxidation/antioxidant system of the body. The components of common intracellular antioxidant systems are described below.

### Xc-GSH-GPX4 system

The Xc-GSH-GPX4 system is a common antioxidant system involved in ferroptosis. It is also known as the cysteine-glutamate reverse transport system and is composed of solute carrier family 7 member 11 (SLC7A11) and solute carrier family 3 member 2 (SLC3A2). It maintains intracellular glutathione (GSH) in a reduced state. GSH is the most important antioxidant in cells and can reduce peroxidized lipids to their corresponding alcohols, thereby reducing the effect of lipid peroxidation. Inhibition of GSH, the reducing substrate of GPX4, is common in ferroptosis. The classical ferroptosis inducer erastin induces ferroptosis by decreasing GSH levels. The catalyst for this reduction is glutathione peroxidase 4 (GPX4), a selenoprotein^109^. The DUBA-SLC7A11-c-Myc axis is essential for ferroptosis resistance in differentiated cancer stem cells (CSCs)^110^. The mechanism of ferroptosis in GPX4 knockout cells is the same as that of erastin-induced ferroptosis^111^. Ten-eleven translocation 2 (TET2), an important demethylase, is an important regulator of lipid peroxidation and iron uptake in airway epithelial cells and can be used as a potential therapeutic target for cigarette smoke-induced chronic obstructive pulmonary disease 112. As an RBM-MKL-1 fusion protein, MKL-1 inhibits ferroptosis in cells. Specifically, MKL-1 expression is increased in gastric cancer tissues, which may be related to the resistance of gastric cancer cells to ferroptosis. Inhibition of MKL-1 enhances the cancer cell-killing effect of ferroptosis inducers such as erastin. Further studies have found that MKL-1 regulates GSH production through the Xc system, which is an important reason for the inhibition of ferroptosis in cancer cells. Upregulation of MKL-1 and SCL7A11 are positively correlated, suggesting that MKL-1 may be a novel target for inhibiting ferroptosis^113^. Cysteine and homocysteine inhibit ferroptosis in a GPX4-dependent manner. Cysteine and homocysteine are GPX4 substrates This pathway is GSH-independent, with GPX4 showing a significantly higher affinity for cysteine and homocysteine than for GSH in the absence of GSH synthetase^114^, suggesting that these two pathways may form a new cellular antioxidant pathway. As a transcription factor that represses antioxidant genes, BACH1 is clinically relevant in active pulmonary tuberculosis. BACH1 deficiency leads to increased glutathione levels and GPX4 expression in the body. *In vivo*, BACH is primarily expressed by alveolar macrophages, neutrophils, and macrophages/monocytes. Moreover, BACH1^-/-^ macrophages are more resistant to Mycobacterium tuberculosis (Mtb)-induced cell death. Prognosis of Mtb infection is significantly improved in BACH1 knockout mice. Mechanistically, BACH1 deficiency is usually accompanied by enrichment of ferroptosis-related genes in alveolar macrophages, neutrophils, and macrophages/monocytes. Taken together, these results demonstrate the importance of BACH in regulating ferroptosis-related genes in different immune cells during Mtb infection^115^. SLC7A11 is also regulated by Activating transcription factor 4 (ATF4). Under endoplasmic reticulum (ER) stress, ATF4 upregulates antiferroptosis genes such as SLC7A11. Therefore, ATF4 may play an important role in maintaining cell viability and preventing lipid oxidation^116^. TET2), a demethylase, has been associated with reduced lung function. Specifically, TET2 regulates cigarette smoke-induced lipid peroxidation via GPX4. Ovarian tumor domain-containing 5 (OTUD5) is highly expressed in triple-negative breast cancer and can affect the sensitivity of triple-negative breast cancer to paclitaxel. OTUD5 stabilizes SLC7A11 in tumor cells by severing the K48-linked ubiquitin chain^117^. TET2 overexpression significantly inhibits ferroptosis. Mechanical studies have shown that TET2 inhibits ferroptosis via demethylation of the GPX45 promoter^118^. Recently, SLC7A11 has been shown to be palmitoylated in glioblastomas, and this modification is required for the stability of SLC7A11. This modification is facilitated by ZDHHC8, a protein palmitoyltransferase (PAT). Consequently, SCL7A11 is not easily degraded by cancer cells, making them less susceptible to ferroptosis^119^. In summary, cancer cells resist ferroptosis by enhancing Xc system activity and intracellular GSH levels, leading to anti-cancer drugs that depend on promoting ferroptosis in tumor cells being rendered ineffective. These findings demonstrate the complex regulation of ferroptosis. Recent studies have shown that dihydroorotate dehydrogenase (DHODH) strongly regulates ferroptosis caused by GPX4 inhibition. Specifically, inactivation of DHODH in GPX4-deficient cancer cells results in mitochondrial lipid peroxidation and ferroptosis. Recent studies have shown that USP18 can significantly reduce the levels of iron ions and the release of MDA, increase intracellular GSH levels, as well as inhibit cell ferroptosis in a mouse model of middle cerebral artery occlusion (MCAO). Some evidence also indicates that GSH negatively regulates ferroptosis^120^. In GPX4-overexpressing cells, DHODH deficiency promotes mitochondrial lipid peroxidation and ferroptosis induced by a ferroptosis inducer. Mechanistic studies indicate that reduction of ubiquinone (CoQ) to panthenol (CoQH2) by DHODH inhibits ferroptosis within the mitochondrial membrane^121^.

### Ferroptosis-suppressor-protein 1(FSP1)

FSP1, also known as inhibitor of ferroptosis 1, is a GPX4-independent protein that inhibits ferroptosis through the FSP1-CoQ10-NADPH axis. Overall, cells do not necessarily undergo ferroptosis when GPX4 alone is inhibited, indicating that cells also inhibit ferroptosis through other pathways, such as FSP1^122^. Functionally, FSP1 can reduce CoQ10 or vitamin K to products such as CoQ10H2 or vitamin K hydroquinone. This suggests that FSP1 acts as an antioxidant that limits intracellular free radical levels and prevents lipid peroxidation. FSP1 has often been described as a downstream gene of p53^123^. It is also a transcriptional target of Nrf2 and PPARα. Interestingly, FSP1 expression is upregulated by Inc RNA expressing MEG3 in T-cell lymphoblastic lymphoma. Thus, FSP1 transcriptionally regulates ferroptosis in cancer cells but also reduces ferroptosis incidence in an NADPH-dependent manner. CD36 expression in the renal tissue of patients with AKI is significantly increased, and its expression is related to renal function. CD36 induces AKI both *in vitro* and *in vivo* by inducing lipid peroxidation in renal cells. Further, the interaction between CD36 and FSP1 has been confirmed using co-immunoprecipitation. CD36 directly binds to FSP1 and degrades it via ubiquitination. This induces ferroptosis in AKI^124^. FSP1 and lipid metabolism-related genes are highly expressed in head and neck squamous cell carcinoma (HNSCC) cells, and in these cancer cells, high FSP1 expression is closely related to tumor resistance. FSP1 knockdown was found to significantly reduced the rate of cancer cell invasion and metastasis^125^. In D-galactose (D-gal)-induced osteoblasts, YBX1 was found to inhibit cell ferroptosis through the m5C-dependent ATF4/FSP1 axis, a potential new target for osteoporosis treatment^126^. In intervertebral disc degeneration (IVDD), FSP1 is involved in activating NF-κB and caspase3-mediated apoptosis, which promotes the development of IVDD. Therefore, FSP1 is likely a new target for treating IVDD^127^. The presence of FSP1 demonstrates the diversity of antioxidant systems and provides new research directions for targeting ferroptosis in disease treatment.

### GCH1-BH4 antioxidant system

A common coenzyme factor involved in intracellular REDOX homeostasis maintenance is BH4 (tetrahydrobioterin), but its role in ferroptosis has only been studied recently. BH4 is synthesized by GTP cycle hydrolase 1 (GCH1), a promising inhibitor of ferroptosis. The GCH1-BH4 axis reduces the risk of lipid peroxidation by depleting ROS, controlling BH4 synthesis, and reducing the oxidized state CoQ to CoQH2 in cells. In particular, it protects its phospholipids and two polyunsaturated fatty acid tails, thus inhibiting the occurrence of ferroptosis. A recent study using CRISPR showed that ferroptosis is controlled by the GCH1-BH4 axis. GCH1 expression is significantly decreased in erastin-treated colorectal cancer (CRC) cells^128^. The sensitivity of CRC cells to the classical ferroptosis inducer erastin is controlled by GCH1. Interestingly, RSL3-induced ferroptosis is not regulated by GCH1. Further, when GCH1 gene expression was decreased, ferritin heavy chain (FTH1) in cells also decreased. FTH1 is regulated by nuclear receptor coactivator 4 (NCOA4)^129^, and it is likely that GCH1 is involved in ferritin-associated autophagy. GCH1 regulates ferroptosis in alveolar macrophages. GCH1 knockout in these macrophages increases the probability of ferroptosis. Moreover, AMPK plays an important role in regulating ferroptosis via GCH1^130^. Taken together, the GCH1-BH4 axis may regulate ferroptosis in cells by limiting both iron uptake and lipid peroxidation. Mesenchymal stem cell-derived exosomes (MSC-Exos) have been found to exert therapeutic effects against acute spinal cord injury (SCI). Specifically, MSC-Exos alleviated microglial ferroptosis by upregulating the Nrf2/GCH1/BH4 axis^131^. Sr-rich splicing factor 1 (SRSF1) plays a key role in the treatment of triple negative breast cancer. SRSF1 has been shown to inhibit GCH1 expression to alleviate cisplatin resistance in breast cancer cells. Therefore, SRSF1 has the potential to be an important therapeutic target in breast cancer^132^. Overall, the GCH1-BH4 axis is a potent ferroptosis inhibitor with a wide scope for development.

### Ferroptosis and ubiquitin

Ubiquitin refers to the modification, regulation, or degradation of target proteins by ubiquitinases. It is an epigenetic regulatory mechanism. These enzymes are involved in protein ubiquitination: E1 ubiquitin-activating enzymes, E2 ubiquitin-conjugating enzymes and E3 ubiquitin-ligase enzymes. The relationship between ferroptosis and E3 ubiquitin ligases has been extensively studied^133^-^135^. E3 ubiquitin ligases regulate ferroptosis as follows. E3 ubiquitin ligases promote downregulation of ASCL4 and reduce the damage caused by lipid peroxidation. Ubiquitination and degradation of p61 by the E3 ubiquitin ligase TRIM21 leads to failure of p61-mediated Nrf2 activation, which reduces the antioxidant capacity of tumor cells and makes them more prone to ferroptosis. The Hippo pathway is also involved in ferroptosis regulation. Thus, the Hippo regulatory mechanism might be related to cell density. E3 ubiquitinases may also be involved in the regulation of Hippo ferroptosis. The downstream Hippo pathway, YAP/TAZ, is activated by tyrosine kinase phosphorylation. E3 ubiquitinase recognizes and degrades phosphorylated YAP, preventing it from continuing downstream signaling resulting in inhibition of ferroptosis^136^. The MARCHF6 E3 Ub ligase is an important regulatory enzyme in ferroptosis. It can promote the degradation of ASCL4 and P53. The carboxy-terminal MRR of MARCHF6 binds directly to the amino terminus of NADPH and E3 ligase, and NADPH is related to the REDOX state of the cell. Therefore, MARCHF6 directly regulates ferroptosis based on intracellular NADPH levels 137. Ubiquitin-specific peptidase 20 (USP20) inhibits ferroptosis in OxA-resistant hepatocellular carcinoma (HCC) cells. USP20-knockout cells are sensitive to OXA, as verified by crystal violet staining. These cells were more sensitive to erastin-induced ferroptosis, with significantly decreased GSH levels, and increased ROS and ferrous ion levels. This effect could be reversed by Ferr-1. We further identified the replication stress signal, ATR, which interacts with USP20. This molecule is also highly expressed in OXA-resistant cells. ATR inhibition results in increased ubiquitination of wild-type USP20. Knockdown of ATR or USP20 reduces the sensitivity of HCC cells to OxA-induced ferroptosis^138^. Ubiquitin-specific protease 5 (USP5) acts as an lymphoid-specific helicase (LSH)-specific deubiquitinase (DUB) in HCC. USP5 overexpression inhibits RSL3-induced ferroptosis in LM3 and HepG2 cells and decreases ASCL4 expression in these cells. By transiently overexpressing LSH after USP5 knockdown, we found that USP5 promoted HCC cell proliferation by stabilizing LSH, and that upregulation of USP5 was associated with poor patient survival^139^. Recently, an E3 ubiquitin protein ligase named TRIM3 was found to promote ferroptosis by increasing ROS accumulation and lipid peroxidation in NSCLC. TRIM3 overexpression significantly improved the prognosis of patients with NSCLC *in vivo*. Mechanistic studies indicate that the NHL domain of TRIM3 interacts with the Xc system, leading to K37 ubiquitination of SCL7A11. This leads to degradation of SLC7A11 and ultimately promotes ferroptosis. Thus, TRIM3 is a potential tumor inhibitor. This is a new and effective strategy for treating NSCLC^140^. Recently, Ubiquitin-specific peptidase 10 (USP10) was found to be associated with poor prognosis in patients with thyroid cancer (THCA). USP10 enhanced THCA cell viability, invasion, and metastasis. Moreover, the levels of MDA, ROS, and free iron ions in THCA cells were reduced, rendering them less prone to ferroptosis. USP10 also increased mitochondrial membrane potential and reduced superoxide production within cancer cells, and its knockdown inhibited THCA cell growth *in vivo*. These results suggest that USP10 is a novel therapeutic target for patients with THCA 141. Overall, the ubiquitin-related protein family may be involved in ferroptosis in cancer cells. Thus, exploring the effects of different ubiquitin-specific enzymes on ferroptosis in tumor cells is a new research direction.

### Epigenetic regulation

Recently, ferroptosis was reported to show a strong relationship with epigenetics. Epigenetic regulation controls the tendency of cells to undergo ferroptosis by regulating the activity of transcription factors and post-transcriptional modifications. Epigenetic regulation of ferroptosis provides a new therapeutic approach for treating cancer and other diseases^142^.

### Non-coding RNA

#### MicroRNA

MicroRNAs (miRNAs) are small non-coding RNAs that act as regulatory RNAs. Their main function is to regulate the expression of various genes in cells^143^. miRNA can change the growth and sensitivity of tumor cells to erastin by regulating ferroptosis^144^. This is one of the mechanisms by which miRNAs regulate the activity of the XC system^145^. For example, MiR-5096 regulates ferroptosis in breast cancer cells by regulating the Xc system and controls ferroptosis by altering ROS content, iron content, and lipid peroxidation levels. Cancer cells overexpressing MiR-5096 show a significantly increased likelihood of ferroptosis^146^. Further, miR-522 has been recently identified as a regulator of ferroptosis. This is because it inhibits the arachidonic acid lipid peroxidase 12 (ALOX15) activity. It reduces PUFA accumulation in cells and downregulates the probability of lipid peroxidation, ultimately preventing ferroptosis. The transfer of MiR-522 to exosomes is regulated by the heterogeneous ribonucleoprotein A1 (hnPNPA1). After subcutaneous injection of CAF cell supernatant with miR-522 knockout into nude mice, the expression level of miR-522 decreased, the levels of ALOX15 and reactive oxygen species in tumor cells increased, the probability of ferroptosis in tumor cells increased, and the number of tumor cells decreased^147^. miR-129-2-3p is significantly upregulated in LPS-treated macrophages. miR-129-2-3p negatively regulates SMAD3, which further promotes macrophage polarization to the M1 phenotype and ferroptosis. Further studies have shown that miR-129-2-3p regulates macrophage polarization and ferroptosis by targeting the SMAD3-GPX4 axis. This novel finding may help treat macrophage polarization in sepsis^148^.

#### lncRNA

Long noncoding RNA (lncRNAs) are a type of ncRNA (non-coding RNA). Although lncRNAs are not directly involved in the synthesis of various proteins in cells, they participate in the regulation of various cellular activities, such as apoptosis, autophagy, tumor development, and necrosis. lncRNAs are associated with cancer occurrence and development. Increasing evidence suggests that lncRNAs are key regulators of abnormal lipid metabolism and siderophore-mediated apoptosis in cancer^149^. lncRNAs are also involved in ferroptosis regulation. By comparing lncRNA arrays, lncRNA-seq was constructed using an RNA library. The results showed that the intraperitoneal metastasis (PM) of GC cells was highly correlated with lncRNA expression. HIF-1α regulates the expression level of SLC7A11 through lncRNA, which reduces the amount of ROS and iron accumulation in gastric cancer cells, and makes them less sensitive to ferroptosis^150^. Moreover, lncRNAs regulate ferroptosis by modulating factors related to mitochondrial oxidation. A classic example is Nrf2. The Nrf2/GABPB1 pathway plays an important role in the oxidative stress response. Metallothionein 1D (MT1DP) promotes erastin-induced ferroptosis in NSCLC cells by increasing the expression of miR-365a-3p and decreasing the expression of Nrf2^151^. The relationship between the prognosis of patients with bladder cancer (BC) and lncRNAs has also been studied. The correlation between the predicted results and the treatment response in patients with BC was analyzed. Some lncRNAs were been confirmed to be closely related to ferroptosis (e.g. OCIAD1-AS1 and AL162586.1)^152^. LINC00618 can downregulate the expression level of SLC7A11 and induce lipid peroxidation and ferroptosis in cancer cells^153^. Nuclear enriched specialized transcript 1 (NEAT1) is a lncRNA that is highly expressed in various human tumors. NEAT1 expression is inversely correlated with tumor growth in human breast cancer cells compared with that in normal breast tissues. However, the expression ofmiR-448 shows and opposite trend to that of NEAT1, and both competitively bind to ZEB1 to regulate tumor growth^154^. A potential mechanism by which NEAT1 regulates tumors is that it indirectly increases the expression of inositol oxygenase (MIOX) by regulating miR-362-3p, thereby increasing the sensitivity of liver cancer cells to ferroptosis^155^. Similarly, PELATON (also known as LINC01272), one of the long non-coding RNA (lncRNA), induces ferroptosis and promotes cancer cell growth by down-regulating mutant p53 in CRC. PELATON was first identified as an lncRNA that inhibited ferroptosis. It reduces ROS production and restricts the entry of iron ions into cells, thereby preventing ferroptosis^156^. p53RRA is a type of cytoplasmic lncRNA, found to be down-regulated in tumor tissues and participating in tumor immunity as a tumor suppressor. p53RRA can also promote the occurrence and development of ferroptosis. p53RRA is involved in activation of the tumor suppressor gene p53^157^. In contrast to p53RRA, another lncRNA, LINC00336, inhibits ferroptosis in lung cancer. Overexpression of LINC00336 limits cell ferroptosis induced by erastin and RSL3. Further, LINC00336 knockout PC9 cells show significantly reduced cell growth and increased sensitivity to ferroptosis^158^, ^159^. The lncRNA LINC00551 is downregulated in tumor tissues compared to that in normal tissues. The prognosis of patients with low LINC0051 expression is reported to be worse than that of the patients with normal expression. After an in-depth study, LINC00551 overexpression was found to significantly reduce cell viability and increase intracellular ROS and Fe^2+^ levels after exposure to the ferroptosis inducer, RSL3. However, treatment with ferroptosis inhibitors such as Ferrostatin-1 (Fer-1) could restore cell viability^160^. DNA damage response (DDR) is known to be involved in ferroptosis occurrence and development^161^. LINC00551 competitively binds miR-4328 and regulates the expression of DNA damage inducible transcript 4 (DDIT4). A positive correlation is found between the expression of LINC00551 and DDIT4. PTPRG-AS1 is a recently discovered lncRNA, and its expression is significantly increased in triple-negative breast cancer (TNBC). Silencing PTPRG-AS1 increased intracellular glutathione reduction and the accumulation of iron ions and ROS in cancer cells. An in-depth study of the mechanism revealed that PTPRG-AS1 targeted miR-376c-3p to upregulate SLC7A11, thereby inhibiting iron deficiency anemia and promoting TNBC development. This suggests that PTPRG-AS1 is a potential target for TNBC treatment^162^. Overall, lncRNAs can regulate ferroptosis to exert their biological functions. Moreover, many unknown lncRNAs remain whose roles in ferroptosis are still remaining to be discovered.

### DNA methylation

DNA methylation is a common mechanism of epigenetic regulation. This regulation typically occurs at cytosine-phosphate-guanine (CpG) dinucleotides. These modifications are often clustered together and referred to as CpG islands. DNA methylation can occur in the promoter regions of genes, leading to gene silencing or deletion. It also plays an important regulatory role in tumor growth^163^, ^164^. DNA methylation induces ferroptosis in tumor cells by regulating GPX4, a key molecule in ferroptosis. These results indicate that DNA methylation is also involved in regulating ferroptosis in tumor cells^165^. Lymphatic-specific helicases (LSH) are closely associated with embryonic development. Loss of LSH leads to pathological changes, such as abnormal DNA methylation, histone tail acetylation, and altered methylation patterns^166^. LSH expression is increased in lung cancer tissues and this alteration makes cells more resistant to ferroptosis. Furthermore, LSH reduces erastin-induced ferroptosis, which is abrogated in LSH-depleted cells. Loss of FADS2 and SCD1, the target genes of LSH, reduces the expression of ferroptosis-resistant factors such as SLC2A4, SLC7A11, and SLC1A5. One of the pharmacological effects of all-trans retinoic acid (ATRA), a classic anti-cancer drug, is to downregulate LSH expression and promote ferroptosis in cancer cells^167^. Runt-related transcription factor 3 (RUNX3) downregulates SLC7A11 expression and induces ferroptosis in gallbladder cancer (GBC) cells through the tumor suppressor gene p53, both *in vivo* and *in vitro*, thereby inhibiting the occurrence and development of gallbladder cancer. DNA methyltransferase 1 (DNMT1) can mediate methylation of RUNX3 to down-regulate RUNX3 in gallbladder cancer cells. RUNX3 downregulation is associated with poor prognosis in patients with gallbladder cancer^168^. Taken together, these results suggest that DNA methylation plays an important role in cancer cell ferroptosis. Thus, DNA methylation can be targeted to develop clinically useful anti-cancer drugs.

### Histone modifications and chromosome remodeling

Histones are parts of chromatin that are often tightly bound to DNA. The lysine residues of histones present the potential for epigenetic modifications, such as methylation, acetylation, and glycosylation. This epigenetic modification often leads to silencing or enhanced gene expression^169^. Epithelial-mesenchymal transition (EMT) has been shown to be closely related to ferroptosis. SET domain-bifurcated 1 (SETDB1) is a histone methyltransferase. We found that expression of GPX4, Nrf2, and HO-1 in SETDB1-knockout cells was significantly decreased, and that the intracellular ferrous ion level was significantly increased. Overexpression of SETDB1 leads to EMT, whereas cells that fail to undergo transformation undergo ferroptosis^170^. KDM3B is a histone H3 lysine 9 demethylase. Previous studies have demonstrated that it prevents ferroptosis in cancer cells. Specifically, KDM3B activated SLC7A11 expression and induced robust resistance to the ferroptosis inducer erastin. KDM3B interacts with ATF4 to activate the SLC7A11 promoter, and lifts the repression of SLC7A11 by p53[^171]^. E1A-associated 300-kDa protein (P300) is a histone acetylase. The activity of P300 in human aortic smooth muscle cells (HASMCs) undergoing ferroptosis is significantly decreased, which is reversed by ferroptosis inhibitor Fer-1. P300 knockout in HASMCs also promotes ferroptosis. By inhibiting the acetyltransferase activity of P300, the acetyltransferase activity of P300 was found to be necessary for resistance to ferroptosis. PVJ\300 regulates ferroptosis by competitively binding HIF-1α to p53 and regulating the expression of heme oxygenase 1(HMOX1)^172^. In acute kidney injury, histone deacetylase 3(HDAC3) mutations can cause GPX4 inhibition, promote ferroptosis of renal epithelial cells, and aggravate AKI development. Further investigation indicates that after arachidonic acid (AA) treatment, HDAC3 binds to Kruppel-like factor 5 (KLF5) in the GPX4 promoter. This results in reduced histone acetylation and inhibition of GPX4 transcription. The reno-protective effect of RGFP966, an HDAC3 inhibitor, is abolished by inactivation of GPX4 in cells treated with RSL3. Overall, HDAC3 inhibition can effectively alleviate AKI^173^. In summary, histone modification plays a significant role in ferroptosis development. Nrf2 mediates epigenetic changes through histone hypermethylation by regulating the production of L-2-hydroxyglutaric acid (L2HG), and generates excessive ROS by reducing the transcription and translation of downstream products, which is involved in the occurrence of ferroptosis in sickle cell disease (SCD). Nrf2 activation effectively improves SCD. These results suggest that targeting Nrf2 is a novel approach for treating SCD^174^. Two WD40 proteins, DCAF8 and WDR76, stabilize LSH during chromatin remodeling. The DCAF8/WDR76/LSH axis effectively regulates the expression of key REDOX genes. This regulation is dependent on DNA hydroxymethylation, which enhances the interactions between WDR79 and LSH, increases DNA oxidation and ROS production, and predisposes cells to ferroptosis^175^. ATAC-seq results showed that Beas-2B cells showed decreased viability, changes in mitochondrial morphology, and susceptibility to lipid peroxidation after exposure to PM2.5. ChIP-qPCR analysis showed that histone modifications, such as H3 lysine 27 acetylation (H3K27ac) and H3 lysine 4 trimethylation, upregulated iron metabolism-related proteins, such as FTH and FTL1, which significantly improved PM2.5-induced asthma in mice. Ferroptosis inhibition ameliorates asthmatic symptoms in mice^176^. The deubiquitinase OTUB1 interacts with the lysine demethylase SET7 to affect the binding activity of OTUB1 to UBC13. Further, SET7-mediated methylation of OTUB1 alleviates the inhibitory effect of OTUB1 on ferroptosis and relieves its effect on erastin-induced intracellular ROS production^177^. Overall, the development of histone modifications to control ferroptosis is a promising approach.

## Ferroptosis and Immunity

Immune factors are involved in the ferroptosis of cells^178^. When other types of cell death are highly immunogenic, we may be able to choose ferroptosis as a key tool for anti-tumor therapy^179^. The role of ferroptosis in the immune response is complex. The effects of ferroptosis on immunity can be classified into four categories.

### Effects of ferroptosis on immune cells

Macrophages are a type of immune cell. They are also involved in innate immunity and antigen presentation^180^. Ferroptosis is closely associated with macrophage function. M1-type macrophages, in particular, have abundant internal iron storage. Macrophages also produce ROS, which kill bacteria^181^. The produced cytokines regulate LOX activity and ferroptosis^182^. KRASG12D promotes macrophage polarization to the M2 type through STAT3 oxidation of fatty acids. KRASG11D activates macrophages and induces polarization via AGER^183^. This is an example of ferroptosis that regulates the immune system. Iron-dependent exosomes in the cytoplasm of cardiomyocytes can significantly increase the expression of the M1 marker NOS2 and decrease the expression of the M2 marker IL-10 in macrophages. This can promote the development of myocardial infarction^184^. The progression of rheumatoid arthritis (RA) is aggravated by ferroptosis in local macrophages. Further studies have shown that M2 macrophages are more sensitive to free iron-induced ferroptosis. The ferroptosis inhibitor liproxatin-1 (LPX-1) alleviates the progression of K/BxN serum transfer-induced arthritis (STIA) in mice while promoting the differentiation of macrophages toward the M2 phenotype. These results suggest that macrophage phenotypic differentiation and ferroptosis are potential therapeutic targets for RA^185^. Tumor-associated macrophages (TAM) are common cells in the tumor microenvironment (TME), and strongly promote tumor cell growth 186. TAMs express significantly higher SLC7A11 levels than those of BMDMs (bone marrow-derived macrophages). xCT, encoded by SLC7A11, is involved in recruiting TAMs and promotes HCC occurrence and development. xCT also upregulates M2 polarization in macrophages, mediated by IL-4^187^. The sensitivity of the same type of macrophages to ferroptosis also differs. For example, BV microglia and PMs are less sensitive to RSL3-induced ferroptosis than PC12 cells, whereas bone marrow-derived macrophages (BMDMs) are very sensitive to ferroptosis^188^. Silver nanoparticles (AgNPs) can induce ferroptosis in macrophages. Specifically, 10 ng of AgNPs can increase the content of the lipid peroxidation marker MDA in RAW 264.7 cells. The intracellular Fe^2+^ content of AgNP-treated cells increases in a dose-dependent manner. AgNPs may thus promote ferroptosis by increasing the labile iron concentration in macrophages^189^. In addition to macrophages, CD8^+^ T cells are closely associated with ferroptosis. IFN-γ released by CD8^+^ T cells can readjust Ascl4-associated ferroptosis. Specifically, phospholipids containing C16 and C18 acyl chains are preferentially integrated with AsCl4-mediated arachidonic acid. This effect is retuned by IFN-γ to induce ferroptosis in tumor cells resulting in anti-tumor effect^190^. Moreover, IFNγ released by CD8^+^ T cells can down-regulate SCL3A2 and SLC7A11 in tumor cells, and promote ferroptosis. Treatment of CD45-ID8 cells with PD-L1 can significantly increase intracellular ROS levels^191^. In CD8^+^ T cell-based studies, Apolipoprotein L3 (APOL3) was found to enhance the tumor-related immune response of CD8^+^ T cells. Increased APOL3 expression inhibited colorectal cancer cell proliferation and promoted ferroptosis. APOL3 simultaneously interacted with L-lactate dehydrogenase A (LDHA). LDHA overexpression is known to inhibit RSL3-induced ferroptosis. APOL3 regulates LDHA stability by regulating its ubiquitination. In addition, APOL3 also regulates the tumor microenvironment in colorectal cancer by regulating the immune activity of CD8^+^ T cells through LDHA^192^. Neutrophils are also closely associated with ferroptosis. In diabetic wound healing, neutrophil extracellular traps (NETs) kill infected bacteria by inducing ferroptosis; however, NETs are also negatively correlated with wound healing^193^. In Abdominal aortic aneurysm (AAA), the most critical pathological process is the injury of abdominal aortic smooth muscle (SMC)^194^. Ferroptosis plays an important role in AAA formation. Specifically, NETs promote lipid peroxidation by up-regulating ASCL4 expression in SMC cells, which is reversed by the ferroptosis inhibitor Fer-1^195^. Moreover, NETs regulate intracellular ferroptosis through the PI3K/AKT pathway, and DNase I knockout can help reverse the downregulation of the PI3K/AKT pathway^196^. Neutrophils from patients with systemic lupus erythematosus (SLE) show typical morphological features of ferroptosis. Ferroptosis is a major driver of neutrophil death in patients with SLE. In SLE, IFN-α induces ROS production within neutrophils in a concentration-dependent manner. This effect is reversed by increasing GPX4^197^. Myeloid-derived suppressor cells (MDSCs) are immunosuppressive cells involved in tumor immunity^198^. The neutral ceramide enzyme N-acylsphingosineamidohydrolase (ASAH2) is highly expressed in MDSCs and is involved in their survival. It inhibits ferroptosis in MDSCs treated with NC06 by inhibiting the p53 pathway^199^. Natural killer (NK) cells in the tumor microenvironment are inhibited by lipid peroxidation, which inhibits their immune function. The activation of Nrf2 can effectively reverse this effect^200^. A previous study has shown that L-kynurenine (L-KYN), a tryptophan metabolite, induces ferroptosis in NK cells in gastric cancer in an AHR-independent manner. This results in a poor prognosis for patients with gastric cancer^201^. Therefore, this provides a promising immunotherapeutic approach for targeting ferroptosis. Similar to NK cells, dendritic cells (DCs) also play an important role in tumor immunity, and their main function is antigen presentation^202^. DCs in tumor tissues have a higher fat uptake rate. Scavenger receptor (SR) plays a major role in lipid uptake by DCs. The function of DCs with high lipid storage is significantly lower than that of normal DCs, and they are more susceptible to ferroptosis^203^. DCs in tumor tissues have a high fat uptake rate. Scavenger receptor (SR) plays a major role in DC lipid uptake. The function of DCs with high fat storage is significantly lower than that of normal DCs, and they are more susceptible to ferroptosis.

### Effect of the immune system on ferroptosis in tumor cells

Ferroptosis plays an important role in tumor immunity. High-mobility group box-1 (HMGB1) is a transcription factor in the human body that plays a role in DNA transcription. HMGB1 can transmit signals during inflammation, leading to the synthesis and release of proinflammatory factors. It can also play a role in ferroptosis^204^. HMGB1 has been shown to regulate erastin-induced ferroptosis via the p38/RAS pathway. HMGB1 also regulates drug resistance in leukemia cells via the p38/RAS pathway. Loss of HMGB1 inhibits lipid peroxidation, providing a new therapeutic strategy for patients with acute myeloid leukemia (AML). HMGB1 also plays a key role in the immune response. Previous studies have shown that HMGB1 can stimulate the release of tumor necrosis factor (TNF) from macrophages with the assistance of TLR4^205^. Some metal cations, such as manganese, can activate the cGAS-STING pathway and promote maturation of DCs. Molybdenum acid manganese nanoparticles (manganese molybdate nanoparticles with polyethylene glycol modification, MMP NDs) can reduce GSH to disrupt the REDOX balance within tumor cells. This increases intracellular ROS and MDA levels, finally inducing ferroptosis. In addition, MMP NDs promote CD8^+^ T cell infiltration. Tumor cell growth is thus restricted in two ways^206^. Further, inhibition of HMGB1 causes dramatic remodeling of the tumor microenvironment in breast cancer cells. Without affecting CD45^+^ cells, HMGB1 inhibition decreases the number of MDSCs and T cells, increases the M1/M2 ratio of macrophages, and increases the number of DCs. These results indicate that the role of HMGB1 in ferroptosis and immunity should not be ignored and further prove that ferroptosis is closely related to tumor immunity^207^. Short-chain fatty acids (SCFAs) like propionate are known to induce ferroptosis In acute myeloid leukemia (AML), propionate induces the apoptosis and ferroptosis of cancer cells. Moreover, propionate increases ROS production and REDOX imbalance, eventually leading to mitophagy. In terms of immunity, propionate increases the immunogenicity of AML cells and this effect promotes DC maturation, which promotes the death of AML cells and improves the prognosis of patients with AML^208^. Different types of immune cells contribute to the ferroptosis pathway in different ways, which can be exploited to harness the effects of ferroptosis by designing drugs that target a wide range of diseases, including cancer.

### Relationship between ferroptosis and immune molecules

Ferroptosis also affects immune molecules in a very complex manner. For example, the ferroptosis activator RSL3 reduces LPS-induced secretion of cytokines such as tumor necrosis factor (TNF) and interleukin-6 (IL 6), as well as limits MCH expression in DCs^209^. Further, lipid peroxidation and elevated iron ion levels have been observed in the synovium of mice with collagen-induced arthritis (CIA), and the ferroptosis inducer RSL3 has been shown to significant joint damage. TNF-protects articular fibroblasts from -ferroptosis, while IL-6 shows the opposite effect. Further studies indicate that TNF can promote cellular cystine uptake, thereby protecting cells from ferroptosis^210^. Similar to TNF, Interleukin-6 (IL-6) is an immune molecule produced in response to noxious stimuli and can rapidly trigger inflammatory responses^211^. In lupus nephritis, some neutrophils show heterogeneity, and some neutrophil-derived IL-6 can transmit signals between B cells and neutrophils. This causes B cells in the lupus kidney to highly express SCL7A11 and thus become resistant to ferroptosis. Inhibition of SLC7A11 accelerates ferroptosis of B cells in lupus kidneys 212. Arachidonic acid enhances the ferroptosis induced by RSL3 treatment^213^. The endoplasmic reticulum stress (ER) response factor X-box binding protein 1 (XBP1) promotes endogenous tumor growth, and is also one of the by-products of lipid peroxidation^214^. XBP1 can activate and induce triglyceride synthesis in DCs, leading to increased lipid synthesis and inhibition of the anti-tumor ability of DCs^215^. Ferroptosis affects the function of immune molecules, thereby interfering with immune function. In contrast, immune factors secreted by cells can promote ferroptosis. This complementary effect may be useful in treating diseases that are currently difficult to treat.

### The impact of ferroptosis on immune checkpoint blockade therapy

Modification of the immune system to suppress tumor development has been studied for decades. Among many immunotherapies, immune checkpoint blockade is the most effective. However, some patients do not respond to this treatment. This is suggested to be a result of the tumor microenvironment^216^. Ferroptosis is a type of immune cell death (ICD). Therefore, we speculate that it may promote immune checkpoint inhibition (ICI)-induced immunotherapy^217^. IFN-γ can be used as one of the cytokines secreted by NK cells and T cells in the tumor microenvironment. IFN-γ, as a pro-inflammatory factor, can up-regulate PD-L1 expression through transcription. PD-L1/PD-1 is believed to inhibit the activity of T cells in the body through exosomes^218^. PD-L1 is a key immune checkpoint regulator^219^. Based on these findings, the relationship between immune checkpoints and ferroptosis is now being explored. Studies based on colorectal cancer treatment have found that ferroptosis induction combined with MDSC inhibition and checkpoint blockade significantly reduce the number of liver metastatic colorectal cancer cells^220^. Inhibition of APOC1 can induce immune activation and enhance sensitivity to anti-PD1 therapy in patients with HCC. Furthermore, APOC1 inhibition can shift HCC TAMs from the M2 to M1 phenotype via ferroptosis^221^. The TYRO3-AXL-MERTK axis is also a key target in ICI therapy. After the TYRO3 axis is activated, it participates in the communication between cancer cells in the tumor microenvironment^222^. AXL inhibition alters the components of the tumor immune microenvironment, such as the number of DCs and CD8^+^ T cell infiltration. Changes in these components are related to changes in myeloid-supporting cytokines. This evidence suggests that AXL also plays a role in tumor immunity^223^. Galectin-1 knockdown in HCC cells can reduce sorafenib resistance, whereas Galectin-1 overexpression had the opposite effect. Galectin-1 regulates the expression of matrix metalloproteinase (MMP)-2 and MMP-9 and causes tumor invasion. Further, galectin-1 upregulation aggravates the decreased sensitivity of HCC cells to sorafenib-induced ferroptosis by reducing lipid peroxidation. Galectin-1 also activates the MET/AXL pathway^224^. TYRO3, a member of the RTK family, is required for tumor cell growth and reproduction^225^. Its expression is associated with prognosis in patients treated with anti-PD-1/PD-L1 antibodies. TYRO3 reduces the therapeutic effects of anti-PD-1/PD-L1 by inhibiting ferroptosis in tumor tissues. Moreover, TYRO3 regulates the ratio of M1/M2 macrophages to promote the formation of the tumor microenvironment^226^. In conclusion, ferroptosis is closely related to immune checkpoints in tumors and other diseases and may exert immune effects by blocking immune checkpoints. Ferroptosis can thus be used to control the immune system and play an effective role in tumor suppression in the future.

### Relationship between ferroptosis and other types of cell death

Similar to ferroptosis, apoptosis is a tightly controlled programmed cell death. It plays an important role in the normal physiological processes of the human body. Apoptosis is a normal response to stimuli, such as infection, injury, and drugs. Apoptosis is also an important mechanism in tumor regulation 227. Ferroptosis and apoptosis are inextricably linked. For example, the tumor suppressor gene p53 regulates apoptosis as well as ferroptosis^228^. γ-Linolenic acid (LA) can be converted to GLA by enzyme D6D. However, GLA inhibits the growth of human neuroblastoma cells. In a C6 glioma rat model, GLA injection increases the frequency of tumor cell apoptosis. PUFA can also induce ferroptosis^229^. Cuproptosis is a newly discovered metal ion-related programmed cell death. Regulators of ferroptosis and cuproptosis show similar mutation frequencies in 33 types of cancers, indicating a strong correlation between ferroptosis and cuproptosis (R = 0.86)^230^. Simultaneously, ferroptosis activators can induce cuproptosis by reducing the intracellular GSH level and inducing cuproptosis in liver cancer cells *in vivo*^231^. Divalent copper ions (Cu^2+^) can induce ferroptosis by promoting GPX4 protein autophagy; however, inhibition of proteasome activity cannot prevent ferroptosis. Notably, Cu does not affect the erastin-induced increase in TFRC and intracellular Fe^2+^ levels, indicating that Cu does not affect cellular iron metabolism^108^. Autophagy is also involved in programmed cell death. Autophagy is an evolutionarily conserved cell death mechanism characterized by destruction of the lysosomal membrane inside the cell, which degrades various biomolecules (such as proteins, RNA, and DNA) inside the cell^232^. Erastin, the classic ferroptosis inducer, has been found to promote cell autophagy. Autophagy-related proteins such as BECN1 and LC3B contribute to erastin-induced ferroptosis in lung fibroblasts. Moreover, erastin does not significantly increase the intracellular iron content in BECN1^+/-^ or LC3B^-/-^ cells^233^. In addition, an antagonistic relationship exists between ferroptosis and pyroptosis, with 3-hydroxy-3-methylglutaryl-CoA reductase (HMGCR) playing a key role in these interactions. Therefore, targeting BRCC36 may be a potential therapeutic option for treating HCC^234^. Ninjurin-1 (NINJ1), a protein that plays a key role in pyroptosis, was recently found to undergo oligomerization during ferroptosis. NINJ1 knockout protects macrophages from ferroptosis-associated plasma membrane rupture (PMR). Furthermore, loss of cell membrane integrity at the early stage of ferroptosis was found to be NINJ1-dependent, and NINJ1 could promote the release of damage-associated molecular patterns (DAMPs). These results suggest that NINJ1 is a key target of ferroptosis^235^. In summary, different types of programmed cell death are closely related to each other, and further studies on this relationship may help deepen the current understanding of ferroptosis.

## Ferroptosis and Disease

### Ferroptosis, cancer, and cancer therapy

**Figure 4:**
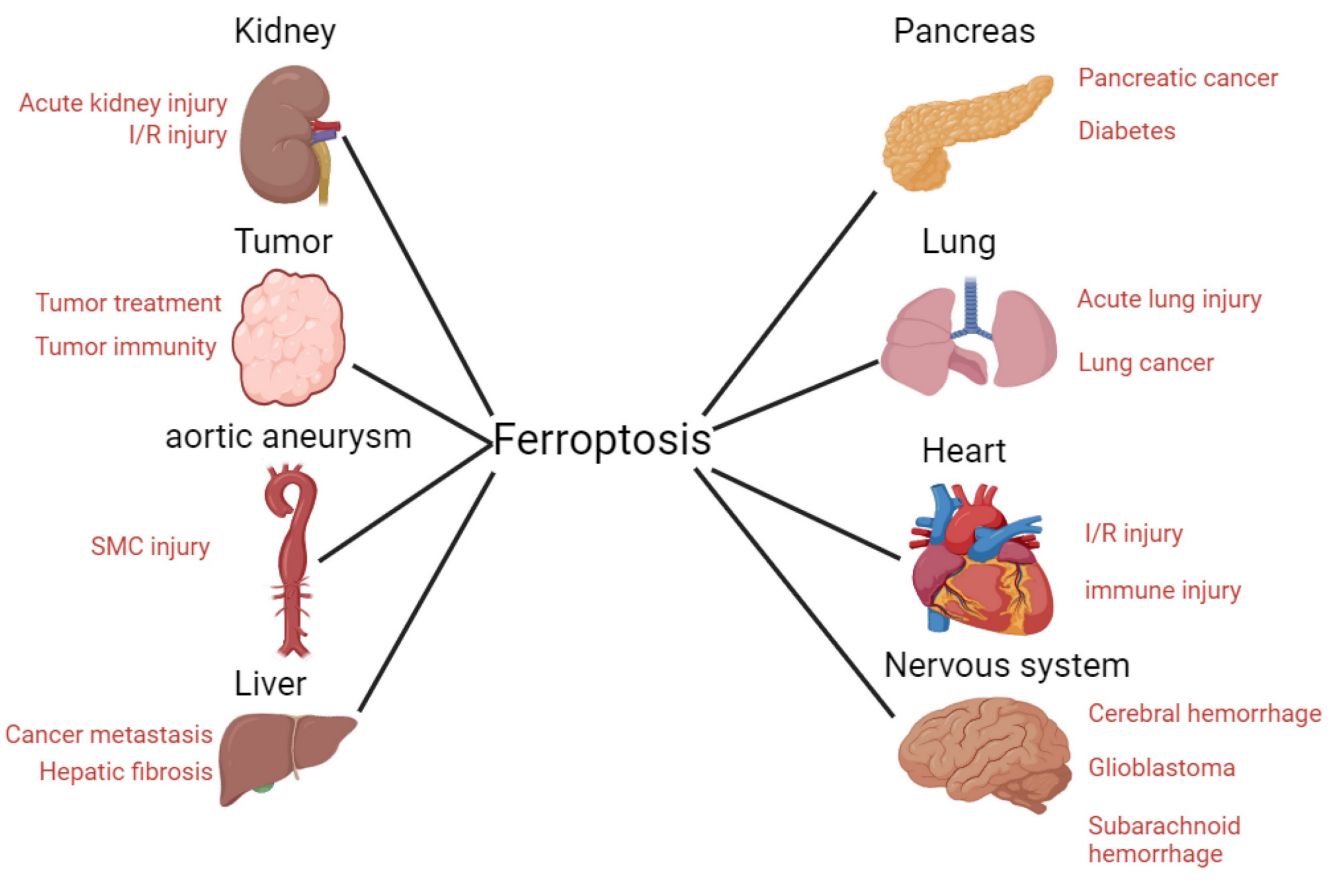
** The relationship between ferroptosis and some diseases:** Ferroptosis is involved in the development of various human diseases, including cancer; it affects multiple organs, including the arteries, liver, lungs, heart, and kidneys.

**Figure 5:**
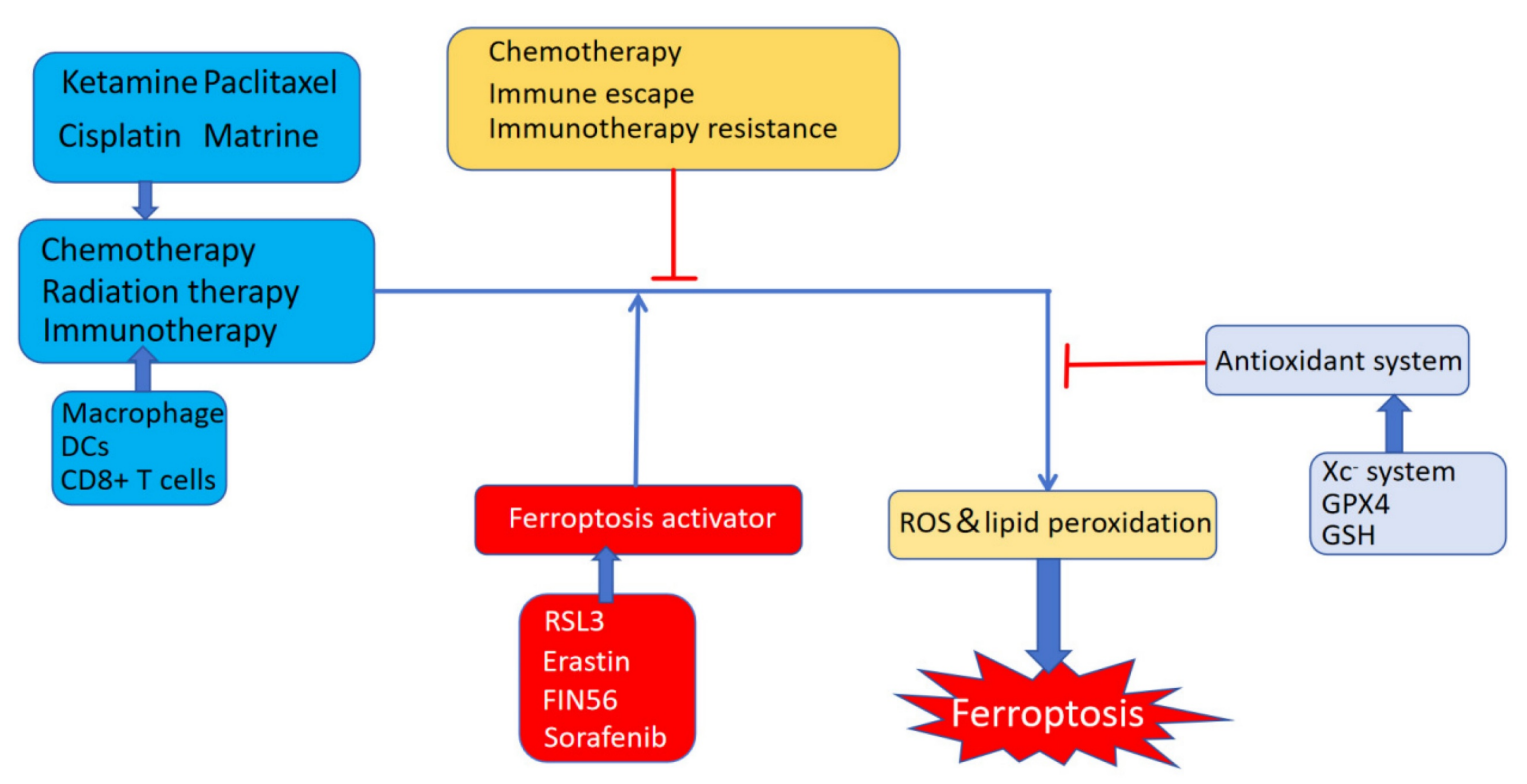
** The relationship between ferroptosis, cancer therapy, and resistance to cancer therapy:** The combination of ferroptosis activators such as RSL3, FIN56, and erastin may have a positive effect on some tumors that are difficult to completely treat using traditional methods. This is because a large proportion of traditional anti-tumor drugs also exert anti-tumor effects by inducing ferroptosis in cancer cells.

Ferroptosis is closely related to the occurrence, development, metastasis, and other pathological manifestations of tumors^236^. Super enhancers (ses) play important roles in the occurrence and development of various malignant tumors (**Figure 4**). Studies have shown that myeloma over-expressed gene (MYEOV) is highly expressed in lung adenocarcinoma cells. Inhibition of MYEOV expression effectively promotes ferroptosis of lung adenocarcinoma cells. Conversely, MYEOV overexpression promotes the proliferation of lung adenocarcinoma cells^237^. A novel endogenous peptide, CTSGDP-13, was found to exert anti-tumor effects in bladder cancer. Its downstream target, TRIM25, significantly promotes ferroptosis in bladder cancer cells and inhibits tumor growth^238^. Cysteine and glycine-rich protein 2 (CSRP2) is upregulated in B cell acute lymphoblastic leukemia (B-ALL). Inhibition of CSRP2 significantly inhibits the proliferation of B-ALL cells and increase their ferroptosis. The transcription factor early B-cell factor 1 (EBF1) is an upstream protein of CSRP2, and its inhibition significantly reduces CSRP2 expression. Overall, the EBF1/CSRP2 axis is a potential therapeutic target for B-ALL^239^. Some tumors such as melanoma may evade ferroptosis via the lymphatic system and blood vessels^240^. Obesity is closely related to the occurrence and development of pancreatic cancer. Studies have confirmed that ferroptosis is more likely to occur in obese patients, and that carcinogenic KRASG12D promotes the development of ferroptosis, which eventually leads to macrophage immunosuppression and the occurrence of first-line ductal adenocarcinoma^241^. Recently, an inseparable relationship has been identified between pancreatic cancer and ferroptosis. Therefore, ferroptosis is a promising target for the treatment of pancreatic cancer. These findings have been used to develop pharmacological and nanotechnological treatments for pancreatic cancer^242^. Drugs targeting ferroptosis are promising candidates for cancer treatment. It can provide a new cure opportunity for many tumors that are poorly treated using (**Figure 5**) traditional methods^243^, ^244^. New nanomaterials have attracted much attention for clinical applications owing to their unique physical properties, such as good electrical conductivity and photoelectrochemical properties^245^. Coating MnO_2_ on Au nanoparticle-anchored black phosphorus nanosheets and subsequent decoration with soybean phospholipids (Au/BP@MS) has been shown to improve electron-hole formation by nanosensitizer separation, which mediates GSH depletion, thereby increasing ROS production in tumor cells; further, MnO_2_ can promote O_2_ production. These two can thus produce a synergistic effect with ultraviolet rays to jointly promote ROS generation in tumor cells; therefore, Au/BP@MS has powerful anti-tumor therapeutic potential^246^. These nanomaterial drugs are good examples, and their invention has proven the potential for clinical translation in ferroptosis research. With the development of new drugs targeting ferroptosis, patient survival will greatly improve in the future. Photodynamic therapy (PDT) is a new method for treating cancer using ferroptosis in recent years. A recent lipid droplet (Ld) targeting type I photosensitizer (PS)produces sufficient O_2_^-^ under light conditions to induce ROS accumulation and ferroptosis in tumor cells, thus achieving anti-tumor effects in hypoxic or conventional environments. Moreover, this photosensitizer exhibits excellent antimetastatic activity^247^. Acidosis has been shown to play a role in breast cancer treatment. The mechanism involves the induction of ferroptosis in breast cancer cells through the zinc finger protein an1 type domain 5 (ZFAND5)/ solute carrier family 3 member 2 (SLC3A2) pathway, increasing the concentration of ROS in tumor cells, and reducing the level of GSH^248^. Currently, the trend in tumor treatment is to produce drugs that are assembled in the patient's body and make them act on the tumor site with high efficiency. For example, recent studies have identified a peptide-iron porphyrin conjugate with specific activation in the tumor microenvironment. The peptide-iron porphyrin conjugate (Gi-F-CAA) is formed by coupling a GPX4 inhibitor to a peptide linker made of iron porphyrin. These complexes can assemble into large nanoparticles (GI-Fs) in the tumor microenvironment. Gi-F significantly inhibits GPX4 activity in cancer cells, increasing the possibility of ferroptosis^249^. PDT is also suitable in combination therapy for cholangiocarcinomas. The clinical efficacy of PDT is often hindered by the tumor microenvironment in cholangiocarcinoma cells. One study combined photodynamic therapy (PDT) with gas therapy to form a CMArg@Lip nanotherapeutic platform. This platform contains Nrf2 inhibitors and NO gas producers. It can induce oxidative damage and aggravate ferroptosis in tumor cells. In addition, CMArg@Lip plays a role in treating cholangiocarcinoma by downregulating PD-L1 in tumor cells and activating the STING signaling pathway^250^. Recently, a novel ferroptosis-inducing agent based on a PEGylated Cu^+^/Cu^2+^-doped black phosphorus@polypyrrole heterojunction (BP@CPP) has been shown to effectively induce ferroptosis in tumor cells. Specifically, BP@CPP enhances ROS production, inhibits GPX4 expression, and accelerates GSH depletion in tumor cells via the Cu^2^⁺ Fenton reaction^251^.

Recently, a novel Cu-doped polypyrrole (CuP) nanoenzyme with trienzyme-like activity has been shown to promote elevation of ROS in the tumor microenvironment and deplete GSH in tumor cells. Moreover, PEGylated CuP nanozymes (CuPP) can act on PD-L1 to enhance the immune response and inhibit tumor growth^252^. Recently, a novel type of liposome was shown to be involved in establishing a new relationship between ferroptosis and tumor immunity. This phospholipid contains two arachidonic acid tails and encloses CpG oligodeoxynucleotides (CpG ODNs). It relies on high levels of metabolism and ROS to recognize cancer cells. Using the standard fluorescent probe 2', 7'-dichlorodihydrofluorescein diacetate, we found that the intracellular ROS level was significantly increased after liposome treatment. Liposomes can help dendritic cells to internalize CpG odn, and simultaneously CpG odn can further promote DC maturation, release a large number of inflammatory factors such as TNF-α and IL-6, and enhance the immune function of CD8^+^ T cells. CpG odn also promotes ferroptosis in tumor cells. These pathways eventually induce tumor growth restriction, and contribute to the anti-tumor effect of liposomes^253^. New materials designed for ferroptosis are currently a focus of research. PROTAC is a semiconductor polymer that limits tumor cell development. This material has a beneficial sonodynamic effect and Fenton reaction characteristics and can produce ROS under stimulation with ultrasound and H_2_O_2_. This effect promotes ferroptosis in tumor cells and delivers PROTAC to tumor cells. This novel nanomaterial inhibits MDSC infiltration and tumor growth in a subcutaneous tumor model in mice^254^. A water-in-oil Lipiodol Pickering emulsion (LPE) coated with calcium carbonate nanoparticles and hemoglobin has been shown to selectively induce lipid peroxidation and ferroptosis in cancer cells based on their hypermetabolic characteristics. These effects could be reversed by ferrostatin or GSH. No significant side effects of LPE were observed in the mouse tumor model. LPE is stable because hemoglobin interacts with calcium ions and improves the hydrophobicity of the LPE surface. The use of LPE in tumor treatment has broad prospects for future clinical application^255^. Currently, several studies have concluded a strong relationship between ferroptosis-related pathways and liver diseases^256^. Breast cancer is one of the many tumors that threaten human health. The lysosome-targeted magnetic nanotorquer (T7-MNT), a new type of lysosome-targeted magnetic nanotorquer, plays an anti-tumor role in breast cancer by effectively inducing ferroptosis in cancer cells. Specifically, lysosome-localized T7-MNT breaks down the lysosomal membrane by generating fluid vortices, resulting in increased lysosomal membrane permeability (LMP). Lysosomal iron spills into the cytoplasm, which causes free intracellular iron ions to increase intracellular ROS levels, thereby increasing the likelihood of ferroptosis. The presence of T7-MNT increases the likelihood of breast cancer being cured^257^. Liver cancer is one of the most common malignant tumors. It is not easily detected in the early stages; therefore, patients with liver cancer are usually diagnosed in the middle and late stages, losing the best treatment time, resulting in poor prognosis. Therefore, the search for drugs to treat liver cancer has become an irresistible trend^258^. Recently, many research teams have attempted to identify new drugs that can induce ferroptosis in liver cancer cells. Ketamine can induce ferroptosis in hepatocellular carcinoma cells *in vitro* and *in vivo*, by reducing the expression of long non-coding RNA PVT1 (lnc PVT1) and GPX4, thereby triggering ferroptosis in hepatocellular carcinoma cells. lncRNA PVT1 binds miR-214-3p and separates it from GPX4. When lncRNA PVT1 expression is decreased, the probability of ferroptosis in liver cancer cells is greatly increased. However, GPX4 overexpression and miR-214-3p inhibition reverse this effect^259^. Ribonucleotide reductase 2 (RRM2), a subunit of ribonucleotide reductase (RRM), plays an anti-ferroptotic role in hepatocellular carcinoma cells. Its mechanism of action mainly involves upregulation of GSH expression, which ensures its interaction with GSS without recognition and degradation by the proteasome via maintained phosphorylation. Therefore, RRM2 may play an anti-ferroptotic role in HCC cells. Dephosphorylation of RRM2 induces ferroptosis in HCC cells^260^. PCBHD14, a member of the cadherin superfamily, plays an anti-cancer role in HCC by inducing ferroptosis in the cancer cells. The promoter can be inactivated by aberrant methylation^261^. Inhibitors of ubiquitin-specific peptidase 8 (USP8) and DUB-IN-3 have shown the most potent anti-cancer activities. Targeting USP8 inhibited HCC proliferation and induced ferroptosis. Specifically, USP8 depletion decreased intracellular cystine levels and glutathione biosynthesis, while increasing ROS accumulation^262^. Polyphyllin I (PPI) showed a dose-dependent inhibitory effect on HCC cell invasion and migration by increasing intracellular ROS and Fe^2+^ levels, depleting GSH, and inhibiting Gpx4 expression. PPI induces ferroptosis and binds to Nrf2, HO-1, and GPX4 proteins. PPI can also damage the mitochondrial membrane and reduce the mitochondrial membrane potential in HCC. Overall, PPI is a potential anti-cancer agent^263^. Induction of ferroptosis is an effective treatment strategy for HCC. Other studies have shown that high levels of m6A modification are closely associated with ferroptosis in HCC. In this process, the reading protein YTHDC2 is an important factor for the increase in m6A levels. YTHDC2 inhibition effectively controls HCC development *in vitro*^264^. In summary, given the high metabolic activity of HCC cells in which lipid peroxidation and iron overload are common, there is a great possibility of developing drugs against ferroptosis. Sorafenib is a novel drug that targets hepatocellular carcinoma (HCC) by inducing ferroptosis. Recent studies have shown that dual-specificity phosphatase 4 (DUSP4) is closely associated with sorafenib resistance in HCC cells. Specifically, the expression of DUSPs, particularly DUSP4, is significantly upregulated in HCC cells following sorafenib treatment. These results suggest that DUSP4 may be associated with sorafenib resistance. Furthermore, DUSP4 knockdown resulted in an interaction between the YTH Domain Containing 1 (YTHDC1) and splicing factor 3(SRSF3), leading to a significant increase in intracellular ROS levels. Further, DUSP4 regulates ferroptosis by regulating FTL expression. The discovery of DUSP4 provides a novel approach to combating sorafenib resistance^265^. In addition to liver cancer, lung cancer poses a major threat to human health. By downloading lung adenocarcinoma samples from The Cancer Genome Atlas (TCGA) and Gene Expression Omnibus (GEO) databases, we determined whether ferroptosis in lung cancer cells is closely related to patient prognosis. Targeting iron-deficiency anemia may be a promising alternative treatment for lung adenocarcinoma^266^. Interestingly, p53 inhibits lung cancer cell growth via the ferroptosis pathway. p53 induces ferroptosis in lung cancer cells by downregulating SLC7A11 expression and upregulating ROS production In contrast, p53 inhibits dipeptidyl peptidase-4 (DPP4) activity to downregulate erastin-induced ferroptosis in a transcription-independent manner. Treatment of Tp53^-/-^ cells with DPP4 inhibitors (such as vildagliptin and alogliptin) completely blocks erastin-induced ferroptosis; however, DPP4 inhibitor treatment was not effective in Tp53^+/+^ cells. Moreover, DPP4 depletion reversed erastin-induced ferroptosis in Tp53^-/-^ cells, indicating that DPP4 is required for ferroptosis in Tp53^-/-^ cells^267^. Lymphoid-specific helicase (LSH) reduces the accumulation of ROS and iron ions by regulating lipid metabolism in lung cancer tissues, ultimately reducing the possibility of ferroptosis in lung cancer cells^268^. GINS4, a eukaryotic G1/S cell cycle promoter, regulates ferroptosis in lung adenocarcinoma (LUAD) cells *in vivo*. Specifically, tumor cells lacking GINS4 are more susceptible to ferroptosis, whereas Fer-1 treatment reverses this effect. In contrast, the levels of ROS and lipid peroxidation significantly decreased, whereas GSH levels increased in GINS4-overexpressing cells. Differential gene expression analysis showed that the anti-ferroptosis effect of GINS4 was related to lipid peroxidation, but not to iron ion levels. GINS4 depletion is particularly sensitive to ferroptosis in G2/M cells^269^. Aryl hydrocarbon receptor (AhR), a member of the transcription factor family, promotes NSCLC development and positively correlates with SLC7A11. AhR binds to the promoter region of SLC7A11, activates expression of SLC7A11, enhances the oxidative sensitivity of cells, inhibits iron function, and promotes NSCLC development^270^. Interestingly, another study on RBMS1 found that its overexpression inhibited the transcription of GPX4 mRNA and promoted ferroptosis. In contrast, RBMS1 prevented SLC7A11 translation. This contradictory effect of RBMS1 may be related to its tissue specificity^271^. FUN14 domain containing 1 (FUNDC1) is a mitochondrial phagocytosis receptor. FUN14 controls the progression of liver fibrosis to HCC by regulating ferroptosis in hepatocytes. Specifically, FUNDC1 ablation significantly ameliorates CCl_4_-induced liver injury. Further studies found that this effect was achieved through regulation of cellular ferroptosis-related factors, such as GSH levels, ROS accumulation, and Gpx4 expression levels in liver cells. Moreover, the protective effect of FUNDC1 knockout against ferroptosis was reversed by mitophagy. This suggests that mitochondrial phagocytosis plays a role in cellular ferroptosis. The effect of FUNDC1 deficiency on ferroptosis is Gpx4-dependent^272^. The occurrence and development of gastric cancer are closely related to ferroptosis^273^, ^274^. Gastric cancer is one of the most common cancers worldwide with a high probability of recurrence after surgery. Therefore, induction of ferroptosis in gastric cancer cells is a promising strategy for treating gastric cancer. CST1, a subtype of cystatin (CST), an inhibitor of cysteine protease, inhibits ROS accumulation in gastric cancer cells when highly expressed. Excessive accumulation of CST can also inhibit the degradation of GPX4 by reducing the ubiquitination level thus reducing the possibility of ferroptosis in gastric cancer cells. Further, CST overexpression increases the metastatic rate of HGC-27 cells^275^. ATF2, a member of the ATF/CEBP family, has been confirmed to be involved in regulating the biological behaviors of gastric cancer cells. Upregulation of ATF2 expression increases the resistance of tumor cells to ferroptosis. Recently, sorafenib was shown to inhibit the growth of gastric cancer cells through ferroptosis. Knockout of the AFP2 gene and combination therapy with sorafenib may be helpful in treating patients with tumors 17. ATF3 is a member of the AFP-CEBP family. ATF3 has been widely studied because of its frequent expression in various tumors^276^. ATF3 expression varies according to the tumor cell type; for example, AFP3 expression is higher in breast cancer^277^; however, its expression level is low in HCC^278^., and prostate cancer^279^. Notably, ATF3 inhibits the growth of gastric cancer cells and relieves their resistance to cisplatin; further its expression level is closely related to the prognosis of patients with gastric cancer. Subsequently, activation of the Nrf2/Keap1 pathway may relieve the inhibition of ATF3 in gastric cancer cells, indicating that ATF3 is likely to cause ferroptosis through Nrf2 to imbalance the antioxidant system in gastric cancer cells^280^. Several tumor suppressor genes, including previously discovered oncogenes like p53, also inhibit tumor progression through ferroptosis^112^. p53 has been reported to play a complex role in tumors. It acts by directly inhibiting glutaminase 2 (GLS2)^281^. STAT6 inhibits p53 acetylation by competitively binding with CBP, thereby restoring SLC7A11 expression^282^. Mouse double-minute 2 (MDM2) is a negative regulator of p53^283^. It is involved in the occurrence and development of many tumors. Examples include breast cancer^284^, HCC^285^, ^286^ and lung cancer^287^. MDM2 functions as a ubiquitin ligase E3, which ubiquitinates p53 for degradation and facilitates the linkage of p53 to the proteasome. Structurally, P2, the second promoter region of MDM2, is activated in a p53-dependent manner, resulting in a negative feedback axis^288^. MDM2 similarly promotes ferroptosis in tumor cells through a p53-independent pathway. Upon comparing the glioblastoma (GBM) high- and low-expression groups for MDM2, the high-expression group was found to be approximately 50-fold more sensitive to RSL3-induced ferroptosis in cancer cells than the MDM2 low-expression group. This effect was reversed by MEL23 with the MDM2 inhibitor^289^. Ferroptosis also plays a role in tumor radiation therapy. Mutant p53 tumor cells are resistant to radiotherapy^290^. After restoring the expression level of p53 in A549 cells, the sensitivity of cancer cells to radiotherapy significantly increased, and significant lipid peroxidation was observed in wild-type p53-expressing A549 cells after adding Nutlin, an inhibitor of MDM2. These results suggest that p53 is involved in radiation-induced lipid peroxidation^291^. Neutrophils in the tumor microenvironment of gastric cancer are prone to ferroptosis, leading the suppression of gastric cancer immunity. Liposomes (LLI) coated with the ferroptosis inhibitor liprostatin-1 and photosensitizer Icy7 were found significantly inhibit the growth of gastric cancer cells. Mechanistic studies indicate that it can enter cancer cells to produce ROS, leading to immunogenic cell death. Simultaneously, liprostatin-1 inhibits the death of neutrophils in the tumor microenvironment. LLI has thus shown great potential in the treatment of gastric cancer^292^. Recent studies have shown that the ubiquitin-specific protease 7 (USP7) inhibitor, DHPO, has a potent growth-inhibitory effect on gastric cancer cells. Mechanistically, DHPO induces ferroptosis in tumor cells by promoting ROS and iron ion accumulation. USP7 regulates ferroptosis of tumor cells by deubiquitination and stearoyl-CoA desaturase (SCD). Taken together, USP7 has high potential in the treatment of gastric cancer^293^. Timosaponin AIII (TA-III), an active ingredient found in plants, enhances lipid peroxidation in colorectal cancer cells to promote ferroptosis. Further studies indicate that TA-III causes mitochondrial damage and excessive ROS production by increasing intracellular fat phagocytosis and promoting the production of free fatty acids (FFA), which eventually lead to cell damage and even death. Rab7 promotes the effects of TA-III on ferroptosis^294^. Matrine induces ferroptosis in cervical cancer cells by activating voltage channels. Compared to the control group, the growth rate of SIHA cells treated with different concentrations of matrine was found to be significantly decreased, and the release of lactate dehydrogenase (LDH) increased. Further studies indicated that matrine could affect Piezo1 (though matrine did not affect the xCT system), and the expression of Piezo1 protein was significantly increased. After Piezo1 knockdown, matrine could no longer promote ferroptosis^295^. Polyphyllin I (PPI) promotes ferroptosis in castration-resistant prostate cancer cells (CRPC) via ERK-DNMT1^296^. The p53 downstream protein, p21, regulates the sensitivity of cells to ferroptosis in tumor tissues. Specifically, after interfering with p21 production in the resistant cell lines HCT-116 and H1299, resistance to ferroptosis decreased. The interactions between p21 and CDKs thus play an important role in the regulation of ferroptosis^297^. Basic helix loop helix transcription factor 40 (BHLHE40) exhibits a strong oncogenic function *in vivo*. BHLHE40 knockdown significantly reduces the metastasis and proliferation of pancreatic cancer cells. Compared to the control group, the tumor volume of BHLHE40 knockout mice was found to be significantly reduced. Further studies have shown that BHLHE40 regulates the transcription of element-binding factor 1 (SREBF1), and that the bHLHE40-SREBF1 axis regulates the protection of pancreatic cancer cells from ferroptosis. Therefore, additional drugs for pancreatic cancer treatment can be developed by targeting BHLHE40^298^. Recently, glutamine has been shown to regulate cellular ferroptosis. Glutamine enters cells via SCL1A5 and SLC38A1^299^. Cysteine depletion in pancreatic cancer cells slows cancer cell growth. Simultaneously, glutamine-starved cells exhibit significantly increased lipid peroxidation and ferroptosis. Further, the use of UHPLC for the isotope labeling of GSSG has revealed that glutamine deprivation makes cells susceptible to ferroptosis by reducing GSH, but not GSSG^300^. The use of ferroptosis in cancer treatment has generally been highly effective, except in melanoma. Recently, a novel liposomal nanomedicine was found to be effective at inducing ferroptosis in melanoma cells. Specifically, the liposomes contained two drugs: the cyclin-dependent kinase 4 and 6 (CDK4/6) inhibitor palbociclib and the ferroptosis inducer auranofin. Liposomes delivered both drugs to the target site with high efficiency. Palbociclib arrested cancer cell growth at the G0/G1 phase, thereby inducing cancer cell senescence and rendering cancer cells more susceptible to auranofin-induced ferroptosis. The combination of these two drugs provides a new therapeutic strategy for targeting ferroptosis and cancer^301^. Gastrointestinal bacteria are closely associated with tumors. Trans-3-indole-acrylic acid (IDA), a tryptophan metabolite derived from the anaerobic bacterium *Peptostreptococcus*, promotes CRC development. It acts on the polycyclic aromatic hydrocarbon receptor (AHR) and up-regulates the expression of ALDH1A3. The latter can be used as a substrate to produce NADH, promote the production of reduced CoQ10, and inhibit ferroptosis. IDA is enriched in colorectal cancer cells. Targeting the IDA-AHR-ALDH1A axis may thus be a novel therapeutic approach for patients with colorectal cancer^302^. In conclusion, ferroptosis plays an important role in tumor development and immunity. Adding ferroptosis-inducing agents to clinical drugs can significantly alleviate the resistance of tumor cells. Ferroptosis is closely associated with drug resistance in tumors during treatment. For example, shikonin effectively inhibited Osteoclast (OC) cell viability when combined with cisplatin. Shikonin shows potent effects against cisplatin-resistant OC cells. The two drugs can up-regulate HMOX1 to increase the accumulation of iron ions in OC cells and induce ferroptosis^303^. Artesunate is a newly discovered drug that exerts anti-tumor effects by promoting ferroptosis in tumor cells. Artesunate conferred resistance to ferroptosis by activating the Nrf2-antioxidant response element (ARE) pathway in head and neck cancer (HNC) cells. Sensitivity of HNC cells to Artesunate was restored by silencing Keap1^304^. Oxaliplatin-induced ferroptosis in tumor cells is also worthy of further investigation. Some studies have reported that the key molecule in oxaliplatin resistance is CDK1, which directly binds to and phosphorylates long-chain acyl-CoA synthetase 4 (ACSL4) and subsequently recruits E3 ubiquitin ligase, causing ACSL4 polyubiquitination, leading to the degradation of ACSL4 protein. In turn, this causes lipid peroxidation and inhibits ferroptosis in tumor cells. This study suggests that for the current ferroptotic drugs that can easily cause drug resistance, resistance of tumor cells to specific drugs can be inhibited by regulating key targets in future studies^305^. The development of drugs targeting ferroptosis in cancer cells thus remains an emerging field.

## Ferroptosis and Sepsis

Sepsis is an organ dysfunction syndrome caused by an imbalance in immune regulation owing to infection, which may lead to death if not treated properly^306^. In patients with septic infection, the common features are suppression of the immune system and massive release of inflammatory cytokines. Some studies have shown that septic cardiomyopathy (SCMs) is closely related to mitochondrial dysfunction. The expression of mitochondria-related GEGs in the immune cell infiltration areas is significantly increased, suggesting that ferroptosis may affect immune cell infiltration of SCMs by regulating mitochondrial function[^56]^. HO-1 plays an important role in sepsis development Further, HO-1 expression was significantly increased in a mouse model of sepsis, with the cells exhibiting typical ferroptotic characteristics. These results suggest that HO-1 is closely related to ferroptosis. Ginsenoside Rb1, an HO-1 inhibitor, effectively inhibits ferroptosis and significantly improves the clinical symptoms of sepsis. Ginsenoside Rb1 has thus been suggested to possess high clinical therapeutic value^307^. DAMPs are released during ferroptosis. Ferroptosis is closely associated with sepsis development. Protein-protein interaction analysis of 34 differentially expressed genes (DEGs) related to iron metabolism revealed that MAP3K5 plays an important role in sepsis and regulates ferroptosis through the JUN pathway. The expression of sepsis-related genes, such as APK14, TLR4, and MAPK8, was significantly increased during ferroptosis. These results suggest that the MAPK pathway plays an important role in sepsis-induced ferroptosis^308^. In the process of bacterial infection, different bacterial species may induce ferroptosis in different ways. For example, *Pseudomonas aeruginosa* uses 15-lipoxygenase (ploxA) to trigger ferroptosis by inducing lipid peroxidation^309^; *Mycobacterium tuberculosis* triggers ferroptosis by reducing the expression of GPX4 in host cells^310^. Inhibition of ferroptosis can greatly reduce various organ injuries (such as heart injury^311^, acute lung injury^312^, and kidney injury^313^).. One such inhibitor is itaconate, which is produced by activated inflammatory macrophages. It reduces ROS production and downregulates lipid peroxidation. Itaconate increases Nrf2 expression and reduces lipopolysaccharide-induced ferroptosis in macrophages. This plays an important role in acute lung injury (ALI). Moreover, Itaconate reduces LPS-induced pulmonary fibrosis, demonstrating its potent protective effects on lung function^314^. Puerarin is used in traditional Chinese medicine. Puerarin protects against sepsis-induced myocardial injury to a certain extent, and this protective effect is reversed by the ferroptosis activator erastin and AMPK pathway inhibitor compound C. These results suggest that puerarin plays a protective role against sepsis by inhibiting the AMPK pathway in myocardial cells thus inhibiting ferroptosis in myocardial cells^315^. FSP1 prevents lipopolysaccharide-induced cell death. Homeobox A5 (HOXA5) is involved in the regulation of AKI occurrence and development in sepsis. In a mouse model of lipopolysaccharide-induced sepsis, HOXA5 overexpression alleviated the clinical manifestations of sepsis. Further, lipopolysaccharide inhibited the binding of HOXA5 to FSP1, an effect counteracted by HOXA5 overexpression. Further studies have shown that SIRT5-induced dessuccinylation of HMOXA5 inhibits lipopolysaccharide-induced ferroptosis by upregulating FSP1 expression 316. Resveratrol (RSV) protects retinal cells from ferroptosis through the Nrf2/GPX4 pathway to maintain retinal nerve function and normal mitochondrial morphology in rats with early diabetic retinopathy. RSV significantly decreases prostaglandin endoperoxide synthase 2 (PTSG2) and MDA *in vitro*. Nrf2 KO cells have been used to verify the molecular mechanisms underlying the inhibitory effect of RSV on ferroptosis^317^. Ginsenoside Rg1 can alleviate sepsis-induced AKI. Ginsenoside Rg1 can reduce ROS levels and iron content in CLP rats. It also increases the levels of GPX4 and FSP1 to protect against ferroptosis. The cytoprotective function of ginsenosides is lost when FSP1 is knocked out; therefore, ginsenoside Rg1 likely exerts its effect by acting on FSP1^318^. The protein and mRNA levels of the RNA-binding protein AUF1 are decreased in alveolar epithelial cells (AECs) after iron stimulation. Ubiquitin ligase E3 regulates AUF1 degradation during ferroptosis. Upregulation of AUF1 in erastin-treated AECs significantly increases cell viability and intracellular glutathione levels. Further studies indicate that Nrf2 protein and mRNA levels are significantly increased in erastin+AUF1 treated cells compared to those in erastin-treated AECs. These results suggest that AUF1 plays an important role in Nrf2 activation and ferroptosis^319^. Melanin nanoparticles (MMPP) can reduce the clinical symptoms of sepsis by preventing ferroptosis of cardiomyocytes in the heart. The ferroptosis marker PTGS2 was significantly reduced in MMPP-treated mice compared with that in control mice treated with LPS to induce sepsis. Moreover, free iron levels in the myocardial cells of the MMPP-treated group decreased, suggesting that MMPP may facilitate resistance to ferroptosis by chelating iron ions. Additionally, MMPP-treated cardiomyocytes exhibited decreased intracellular ROS levels, decreased LDH release, and higher cell viability. These results suggest that MMPP inhibits ferroptosis in cells in many ways. Therefore, MMPP is a promising and clinically effective drug for the treatment of sepsis^320^. Melittin, a natural anti-inflammatory agent, can protect the kidney tissue from sepsis-induced damage. Further studies have shown that this protective effect is achieved by inhibiting ferroptosis, promoting Gpx4 expression and promoting the nuclear transformation of Nrf2 in kidney cells^321^. Ferroptosis is one of the reasons for the reduction in the number of monocytes in the body during the development of sepsis. Glycosylation of zrt-/irt-like protein 8(ZIP8), encoded by SLC39A8, is significantly increased in monocytes from patients with sepsis. Down-regulation of ZIP8 alleviates lipopolysaccharide (LPS)-induced lipid peroxidation^322^. YAP1 in the Hippo pathway has been shown to alleviate lipopolysaccharide-induced ferroptosis and to significantly reduce ROS accumulation and intracellular free iron ion level in MLE-12 cells during sepsis-induced acute lung injury. YAP1 overexpression also blocked lysosomal ferritin degradation. Further studies revealed that YAP1 inhibits ferroptosis by interfering with the NCOA4-FTH1 interaction to inhibit ferritin degradation. Therefore, conditional knockout of YAP1 can aggravate sepsis-induced acute lung injury^323^. In conclusion, ferroptosis is not only involved in the occurrence and development of sepsis but also plays an important role. The application of ferroptosis-inhibiting drugs to control sepsis is a promising research direction.

### Ferroptosis and nervous system diseases

Diseases of the nervous system include Alzheimer's disease, glioma, and cerebral hemorrhage. Ferroptosis is inextricably linked to neurological diseases^324^. The expression of various ferroptosis-related genes (such as Nrf2, GPX4, and FSP1) in different regions of the brain at different times suggests that ferroptosis occurrence and development are related to the development and function of the nervous system^325^. During the development of the juvenile human brain, adequate iron is required to maintain development. However, excessive iron can increase the risk of ferroptosis in nerve cells; therefore, nerve cells require a relatively complete iron regulatory system to maintain iron balance in the brain regions. TAZ/YAP of the Hippo pathway play a role in Schwann cell development and participate in myelin sheath formation. Laminin and mechanical stimulation regulate TAZ/YAP in Schwann cells^326^. As humans age, accumulation of iron in the body may become a key factor in ferroptosis of the nervous system owing to a decrease in iron requirement, aging, and death of red blood cells^327^. Previous studies have attempted to use drugs that inhibit ferroptosis to treat nervous system disorders. For example, cepharanthine can reduce brain injury after a subarachnoid hemorrhage (SAH). Specifically, cefotenine can alleviate m2 ferroptosis of endothelial cells and microglial cells after SAH by inhibiting lipoxygenase-15 (ALOX15)^328^. Baicalin can reduce early brain injury (EBI) after SAH via inhibition of autophagy-dependent ferroptosis after SAH by reducing the levels of malondialdehyde (MDA) and ROS^329^. In summary, the role of ferroptosis in neurological disease development remains unclear, and further research is required to develop drugs that target ferroptosis. Glioblastoma is a serious type of brain tumor. Glioblastomas are resistant to ionizing radiation largely because of the upregulation of internal factors that antagonize ferroptosis, such as Nrf2. Src kinase plays a key role in this process. Inhibition of Src kinase activity promotes Keap1-Nrf2 interaction and Nrf2 ubiquitination for degradation. Glioma cells are prone to ferroptosis. Src kinase also promotes the expression of p62, which competitively binds to Keap1 and attenuates Nrf2 ubiquitination. Therefore, Src kinase may be a potential therapeutic target for glioblastoma treatment 330. Recent studies have shown that PAR1 inhibition alleviates ferroptosis after peripheral nerve injury (PNI) in rats through the Hippo-YAP/ACSL4 pathway. The PAR1/Hippo pathway is thus expected to be a new target for treating peripheral nerve injury^331^. The main causes of brain diseases are neuronal damage and diabetes-related cognitive dysfunction. Hippocampal neurons are the most susceptible sites of ferroptosis in the brain. The CA3 region is particularly affected by ferroptosis. Liproxstatin-1, a ferroptosis inhibitor, significantly improved the number and shape of neurons in the CA3 region of the brain compared to those in the diabetic group. Furthermore, treatment with AICAR, an AMPK agonist, significantly improved cognitive performance and increased GPX4 expression in the mouse brain. Further, lipocalin 2 (LCN2) was significantly increased in the hippocampus of diabetic mice, and its expression was weakened after AICAR treatment, suggesting that LCN2 may be involved in the development of ferroptosis in the hippocampus of mice^332^.

### Ferroptosis and acute kidney injury

Acute kidney injury (AKI) is a common and rapidly developed kidney disease^333^. Many factors can lead to acute kidney injury, including ischemia-reperfusion injury^334^, infections^335^ and drugs^336^. Accumulating evidence suggests a close association between acute kidney injury and ferroptosis. The role of ferroptosis in AKI differs. For example, ASCL4 expression is increased in injured renal cells during I/R-induced AKI. Increased COX2 expression and decreased GPX4 expression have been detected by western blotting, accompanied by mitochondrial structure changes^337^. One of the proteins of the ATP release pathway, pannexin (Panx1), belongs to the DAMP family. It also regulates ATP release. Previous studies have demonstrated that inhibition of Panx1 decreases MDA levels in I/R-induced AKI^338^, In AKI caused by rhabdomyolysis, destruction of striated muscle cells leads to the release of large amounts of myoglobin into the blood, which is the main factor leading to kidney injury^339^. Myoglobin in the kidney is degraded to produce free iron, which can undergo the Fenton reaction accompanied by the upregulation of ptgs2 and Acsl4, eventually leading to renal cell ferroptosis^340^. Heme oxygenase-1 (HO-1) metabolizes heme to bilirubin or biliverdin. It plays a key role in ferroptosis of renal cells. Many factors control the expression of HO-1, including HIF-1α and Nrf2^341^. Application of hepcidin (an iron homeostasis regulator) to improve AKI by controlling the homeostasis of endogenous iron is expected to improve patient survival 342. GPX4 in kidney cells causes AKI in a ferroptosis-dependent manner Studies have shown that GPX4 knockout in mice can induce AKI^343^. HO-1 expression in renal cells is increased in diabetic mice. The ferroptosis inhibitor Ferrostatin-1 can reduce HO-1 expression in renal cells, thereby reducing renal injury^344^. Cisplatin injection induced AKI in wild-type C57BL/6 mice. Immunohistochemistry (IHC) showed that 4-HNE, a ferroptosis marker, was significantly increased in mouse kidney cells, accompanied by a decrease in GPX4 expression, however, the ferroptosis inhibitor Ferr-1 could alleviate AKI^345^. The addition of paricalcitol, a Vitamin D receptor (VDR) agonist, was found to improve renal function and reduce 4-HNE accumulation. Analysis of mouse and human GPX4 promoters using the JASPAR database revealed that VDR could also act as a transcription factor regulating GPX4 expression^340^. Folic acid (FA) is a water-soluble vitamin associated with AKI. Low doses of FA are reported to inhibit ROS production and play a protective role in the kidney^346^. However, high doses (a single intraperitoneal injection of 250 mg/kg) of FA can also cause AKI in mice, and continuous injection can cause AKI to convert to chronic kidney disease (CKD) in mice^347^. The mechanism of FA-induced AKI is likely to increase the levels of ROS in renal cells and decrease the levels of mitochondrial glutathione protein. N-acetyl-cysteine (NAC) reduces the concentrations of NADPH and L-arginine (the precursor of peroxides) in the renal cortex^348^, reducing the possibility of ferroptosis in renal cells. Gastrodin (GAS) is an active ingredient extracted from the plant *Gastrodiae gastrodiae*, and can be used to treat kidney injury. GAS protects HK-2 cells by inhibiting free radical generation and lipid peroxidation^349^. Recently, a novel compound, a 3-hydroxypyridin-4 (1H)-1 derivative, was found to exert antiferroptotic activity. This novel derivative significantly reduced the erastin-induced increase in the intracellular labile iron pool (LIP) detected using the Fe^2+^ probe FeRhoNox-1. Further, ROS and lipid peroxide levels were reduced as indicated by the DCFH-DA and BODIPY-C11 probes, respectively, suggesting that the novel derivative had a potent anti-ferroptosis effect. This compound could reduce cisplatin-induced cell damage. As cisplatin often induces AKI, this compound is likely to become a commonly used drug in clinical practice to reduce AKI symptoms^350^. Ferroptosis is an important cause of nephron reduction in AKI. In this process, the appearance of platelet-activating factor (PAF) and PAF-like phospholipids (PAF-Lpls) leads to cell membrane instability, which in turn leads to ferroptosis induced by lipid peroxidation. This affects the surrounding cells. Addition of PAF-acetylhydrolase (II) (PAFAH2) delays ferroptosis onset, and knockout of PAFAH2 increases PAF production and aggravates AKI-induced ferroptosis and ischemia/reperfusion injury^351^. Deficiency of sulfide:quinone oxidoreductase (SQOR), a mitochondrial inner membrane protein highly expressed in the renal cortex, aggravates cisplatin-induced renal tubular epithelial cell injury and induces ferroptosis. These findings suggest that SQOR may play a role in protecting renal tubular epithelial cells from ferroptosis during AKI. In the AKI mouse model with SQOR overexpression, serum creatinine (SCr), blood urea nitrogen (BUN), and other indicators of kidney injury were significantly lower than those in control mice. SYVN1 is an E3 ubiquitin ligase that mediates SQOR degradation Therefore, the SYVN1-SQOR axis is likely to play an important role in AKI^352^. Ferroptosis may thus be an important factor in regulating cell death during AKI development, and new drugs targeting renal cell ferroptosis to inhibit AKI are expected to be discovered in the future.

## Conclusions

In summary, ferroptosis in diseases has received considerable attention because of its unique biochemical mechanisms. An in-depth study of ferroptotic diseases has revealed that its regulatory mechanisms are complex. The core features of ferroptosis are the accumulation of intracellular iron ions and the occurrence of lipid peroxidation. When various factors (such as a REDOX imbalance) lead to the release of a large amount of Fe^2+^ from the unstable internal iron pool of the cell, Fe^2+^ leads to a large Fenton reaction within the cell and produces excessive ROS. These ROS attack the PUFAs on the cell membrane, leading to the occurrence of lipid peroxidation and eventually leading to ferroptosis of cells. The currently known inducers of ferroptosis include erastin and iron nanomedicines such as MIL-101(Fe) NPs as well as common anti-tumor drugs such as paclitaxel. Many ferroptosis treatments have been reported in clinical practice, for cancer, urinary system diseases, and nervous system diseases. These cases illustrate the broad prospects for the clinical application of ferroptosis. In the current clinical studies of ferroptosis, ferroptosis agonists are not very specific, and some ferroptosis agonists can promote normal cells to undergo ferroptosis, resulting in drug-related side effects. The mechanism of ferroptosis is still under investigation, and some of the mechanistic studies described in this review need to be translated into clinical practice as soon as possible. Existing research has greatly improved the clinical prognosis of patients with tumors and other types of diseases. However, some shortcomings still remain in this review. First, the provided description of ferroptosis-related pathways is not exhaustive, and current research may still need to explore drugs that target regulation of the ferroptotic pathway to prove their important role in the process. Second, sufficient evidence to prove the correlation between certain diseases and ferroptosis remains lacking, which is a key point for future research. Currently, there are few drugs that can induce ferroptosis, and these have significant side effects. Therefore, development of specific drugs (e.g., nanomedicines) that can effectively target ferroptosis is needed. Future clinical research should thus focus on the development of ferroptosis inducers and extend the role of ferroptosis. Effective control of disease occurrence and development by regulating ferroptosis also remains a promising research hotspot.
